# Insight into the atomic-level structure of γ-alumina using a multinuclear NMR crystallographic approach†

**DOI:** 10.1039/d5sc01198a

**Published:** 2025-03-12

**Authors:** M. Bonifac Legrady, Daniel M. Dawson, Paul B. Webb, Sharon E. Ashbrook

**Affiliations:** a School of Chemistry, EaStCHEM and Centre of Magnetic Resonance, University of St Andrews North Haugh St Andrews KY16 9ST UK sema@st-andrews.ac.uk

## Abstract

The combination of multinuclear NMR spectroscopy with ^17^O isotopic enrichment and DFT calculations provided detailed insight into both the bulk and surface structure of γ-Al_2_O_3_. Comparison of experimental ^17^O NMR spectra to computational predictions confirmed that bulk γ-Al_2_O_3_ contains Al cations primarily in “spinel-like” sites, with roughly equal numbers of alternating Al^VI^ and Al^IV^ vacancies in disordered “chains”. The work showed that overlap of signals from O^IV^ and O^III^ species complicates detailed spectral analysis and highlighted potential problems with previous work where structural conclusions are based on an unambiguous assignment (and quantification) of these signals. There was no evidence for the presence of H, or for any significant levels of O vacancies, in the bulk structure of γ-Al_2_O_3_. Computational predictions from structural models for different surfaces showed a wide variety of protonated and non-protonated O species occur. Assignment of signals for two types of protonated O species was achieved using variable temperature CP and TRAPDOR experiments, with the sharper and broader resonances attributed to more accessible surface sites that interact more strongly with water and less accessible aluminols, respectively. DFT-predicted ^1^H NMR parameters confirmed the ^1^H shift increases with denticity but is also dependent on the coordination number of the next nearest neighbour Al species. Spectral assignments were also supported by ^1^H–^27^Al RESPDOR experiments, which identified spectral components resulting from μ_1_, μ_2_ and μ_3_ aluminols. Combining these with ^1^H–^27^Al D-HMQC experiments showed that (i) μ_1_ aluminols are more likely to be bound to Al^IV^, (ii) μ_2_ aluminols are coordinated to all three types of Al, but with a higher proportion bound to similar types of Al and (iii) μ_3_ aluminols are most likely bound to higher coordinated Al species. ^1^H DQ MAS spectroscopy confirmed no aluminols exist exclusively in isolation but showed that the closest proximities are between bridging aluminols coordinated to Al^IV^ and/or Al^V^ species.

## Abstract 2

Insight into the detailed bulk and surface structure of γ-Al_2_O_3_ is provided by a NMR crystallographic approach, exploiting isotopic enrichment, multinuclear experimental measurements and periodic calculations.

## Graphical abstract

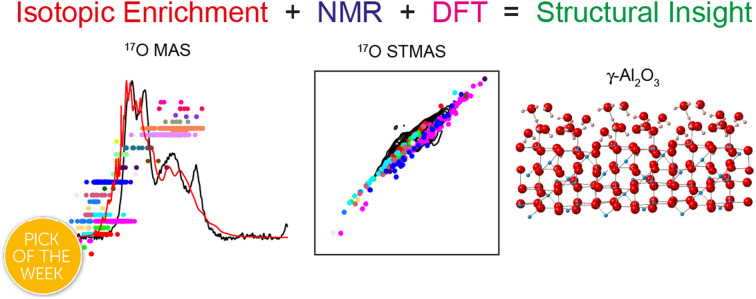

## Introduction

Transition aluminas (Al_2_O_3_) are among the most widely used oxides in heterogeneous catalysis.^1^ Alumina is employed as a support material for processes including methanol production and Fischer–Tropsch synthesis, and can be used to catalyse transformations such as alcohol dehydration, skeletal isomerisation or the removal of sulfur from gases in the Claus process.^2–6^ The versatility of aluminas can be attributed primarily to a high mechanical and chemical stability, high surface area and porosity, and the Lewis-acidity of coordinatively unsaturated surface Al sites. Yet despite their industrial importance, the atomic-scale surface, sub surface and bulk structure of these materials is not well understood.

There are many alumina polymorphs or “forms”,^7^ typically denoted by Greek letters, with the most thermodynamically stable being α-Al_2_O_3_. Aluminas can be categorised into two types based on the arrangement of oxygens in the structure, with a hexagonal close packed (hcp) array for the α, κ and χ forms and a face centred cubic close packed (fcc) array for the η, γ, δ and θ forms.^4,7–11^ The distinct forms are then further distinguished by different arrangements of the Al cations. γ-Al_2_O_3_ is one of the most studied transition aluminas, yet its precise structure is still the subject of considerable and ongoing debate, with several different models proposed in the literature.

γ-Al_2_O_3_ is often described as a non-stoichiometric or defect spinel,^12,13^ with a cubic unit cell that contains cation vacancies to maintain charge neutrality, although consensus on the preferential location of the vacancies (*i.e.*, on tetrahedral or octahedral sites) and on any clustering or ordering has yet to be reached. A number of computational studies determined it was energetically most favourable to locate vacancies on the octahedral (Al^VI^) sites with maximum separation between them.^11,14–20^ However, other authors concluded from molecular dynamics (MD) simulations that cation vacancies are located at tetrahedral (Al^IV^) sites,^21^ while Lee *et al.* suggested vacancies were present on both types of sites (∼63% Al^IV^ and ∼37% Al^VI^).^22^ Many experimental studies have also tried to resolve this controversy, with results from X-ray diffraction (XRD), electron microscopy and ^27^Al NMR spectroscopy suggesting vacancies were located exclusively on Al^VI^ sites,^23–28^ while other electron diffraction and NMR studies have been interpreted as evidence for vacancies occurring at Al^IV^ sites.^29–33^ Some diffraction-based studies have also reported tetragonal distortions of the cubic unit cell.^13,29,34,35^ It has also been suggested from diffraction data that Al^3+^ cations could occupy non-spinel interstitial positions (although there is also some debate over whether these are Al^IV^ or Al^VI^ sites),^36,37^ while work from Paglia *et al.* (using primarily diffraction, microscopy and computation) proposed a tetragonal model for bulk γ-Al_2_O_3_, with over 40% of the Al^3+^ cations occupying Al^VI^/Al^IV^ non-spinel sites.^35^ A different non-spinel model, derived from MD simulations and exhibiting a monoclinic unit cell was described by Krokidis.^38^

Surprisingly few detailed NMR studies have been carried out on γ-Al_2_O_3_, likely reflecting the challenges associated with highly disordered materials.^39^ The majority that have been performed have largely focussed on ^27^Al NMR, drawing conclusions from the relative intensities of the Al^IV^ and Al^VI^ signals in magic-angle spinning (MAS) NMR spectra.^38,40^ Although the ^27^Al chemical shift depends strongly on coordination number, the broadening arising from the quadrupolar interaction, and as a result of disorder, often limits the extraction of precise parameters, particularly at the lower B_0_ field strengths used in earlier work.^39^ It has also been shown that the ^27^Al MAS NMR spectrum can vary with the methods used for sample preparation, the particle size, surface area and hydration state.^38,40^ However, the nature of the cation site (*i.e.*, spinel or non-spinel) is not easily determined from the ^27^Al NMR spectrum (as the immediate local environments in these two are very similar), with cation vacancies occurring only in the second coordination sphere, resulting in a more minor variation in the NMR parameters through small changes in the positions of the neighbouring O^2−^ anions. In contrast, the local geometry around the O^2−^ anions changes significantly with neighbouring cation vacancies, and the arrangement of the surrounding Al^3+^ cations depends crucially upon whether they occupy spinel or non-spinel positions in the structure. Given the low natural abundance of the NMR-active ^17^O isotope (0.037%), and the cost and practical challenges associated with isotopic enrichment,^41^^17^O NMR spectroscopy of alumina has been much less common,^42–47^ although early work was performed by Walter and Oldfield (as discussed later).^42^ Perhaps of most note, is recent high-field NMR work using double-quantum NMR spectroscopy to identify different types of surface and sub surface species, which indicated a non-random distribution of O species, but used the earlier assignment of the signals proposed by Walter and Oldfield.^43^

In this work we combine multinuclear (^1^H, ^17^O and ^27^Al) and multidimensional NMR spectroscopy of γ-Al_2_O_3_ with periodic planewave density functional theory (DFT) calculations of potential structural models of the bulk and surface structure to gain insight into the atomic-scale structure of this important, yet poorly understood, material. This NMR crystallographic approach is vital for interpreting the spectroscopic signals observed and showing that these can often result from an overlap of resonances from different crystallographic environments, which has hindered spectral assignment in previous work. We show that ^17^O NMR spectroscopy is a sensitive probe of the detailed local structure, and of the surface structure in particular, providing, when combined with computation, one of the most successful approaches to date for structural characterisation of this industrially important material.

## Methodology

### Synthesis and isotopic enrichment

Commercial boehmite-derived γ-Al_2_O_3_ was obtained from Sasol UK Ltd (brand name Puralox) and used either as received, after dehydration or after ^17^O enrichment. Samples were dehydrated at 150–300 °C by heating 150 mg in a quartz vial in a sand bath on a hotplate. For dehydration at higher temperatures 100–200 mg of sample was transferred into a quartz vial and heated under vacuum at 500 or 700 °C for 12–24 h in a tube furnace. After dehydration, samples were packed into commercial ZrO_2_ rotors in a glovebox under an inert N_2_ atmosphere. Isotopic enrichment was carried using post-synthetic exchange with 70% ^17^O_2_ (g). Approximately 150 mg of sample was transferred into a quartz vial and immersed in liquid N_2_, before exposure to ^17^O_2_ (g), which then condensed in the pores. The vial was then returned to room temperature, placed in a tube furnace and heated at a temperature between 500 and 950 °C for 12–72 h. Samples isotopically enriched at *X* °C will be referred to as γ-Al_2_^17^O_3_(*X* °C) throughout. Although the absolute level of enrichment was not measured directly, this is estimated to be ∼25–30% by comparison to materials with known enrichment levels.

### NMR spectroscopy

Solid-state NMR spectra were acquired either in house using Bruker Avance III instruments equipped with 9.4 or 14.1 T wide-bore magnets, at the Scottish High-field NMR facility using a Bruker Avance NEO spectrometer equipped with a standard-bore 18.8 T magnet, or at the UK High-Field NMR facility using a Bruker Avance III spectrometer equipped with a 20.0 T wide-bore magnet. Powdered samples were packed into ZrO_2_ rotors and rotated at MAS rates of 5–40 kHz or using conventional double- or triple-resonance probes. NMR spectra are shown referenced relative to H_2_O(l) for ^17^O, 1 M Al(NO_3_)_3_ (aq) for ^27^Al (determined using a secondary reference of aluminium acetylacetonate (*δ*_iso_ = 0 ppm)) and TMS for ^1^H (determined using a secondary reference of l-alanine (*δ*(N**H**_3_^+^) = 8.5 ppm)). Spectra were acquired at ambient temperature unless otherwise stated. High-resolution NMR spectra were acquired using MQMAS^48^ (^27^Al) and DQF-STMAS^49,50^ (^17^O) experiments. For more detailed experimental parameters see figure captions and Section S1 in the ESI.†

### Computation

Periodic planewave DFT calculations were carried out using the CASTEP code (version 16.12).^51–53^ Calculations were performed using the PBE exchange correlation functional^54^ and the semi-empirical dispersion correction scheme of Tkatchenko and Scheffler.^55^ Core-valence interactions were described by ultrasoft pseudopotentials, accounting for scalar relativistic effects using ZORA.^56^ Planewave energy cutoffs of 45 or 60 Ry were used for geometry optimisation, and 60 Ry for all NMR calculations, with the first Brillouin zone sampled using a Monkhorst–Pack grid^57^ with a reciprocal space grid spacing of 0.04 2π Å^−1^ in all cases. Initial structural models by generated by introducing vacancies into an undistorted (Al_3_O_4_) spinel structure and modified as described below. In the geometry optimization all atomic coordinates and unit cell parameters were allowed to vary for bulk models, while unit cell parameters were constrained for the surface models. Further models of bulk and surface structures were generated as described in the text and Sections S3–S6 of the ESI.†

NMR parameters were calculated using the gauge-including projector augmented wave (GIPAW^51^) approach to reconstruct the all-electron wavefunction in the presence of a magnetic field. Calculations provide the absolute shielding tensor (*σ*), *J* coupling tensor (*J*) or electric field gradient tensor (*V*). Diagonalization provides their respective principal components, where *σ*_11_ ≤ *σ*_22_ ≤ *σ*_33_, *J*_11_ ≤ *J*_22_ ≤ *J*_33_ and |*V*_*xx*_| ≤ |*V*_*yy*_| ≤ |*V*_*zz*_|. The isotropic shielding is given by *σ*_iso_ = (1/3)Tr(*σ*) and the predicted isotropic chemical shift by *δ*_iso_ = *σ*_ref_ – *σ*_iso_. Reference shieldings (*σ*_ref_) were determined by comparing experimental shift and calculated shielding for α-Al_2_O_3_ (^27^Al and ^17^O) and TMS (^1^H).^42,58^ The quadrupolar coupling constant, *C*_Q_ = *eQV*_*ZZ*_/*h* was obtained using nuclear quadrupole moments of 146.6 and −25.58 mb, for ^27^Al and ^17^O, respectively.^59^ From the quadrupolar magnitude, *C*_Q_, and asymmetry, *η*_Q_ = (*V*_*XX*_ – *V*_*YY*_)/*V*_*ZZ*_, the quadrupolar product *P*_Q_ = *C*_Q_ (1 + *η*_Q_^2^/3)^1/2^ can be determined. The isotropic *J* coupling is given by (1/3)Tr(*J*).

Density matrix simulations were performed using SIMPSON.^60^ Details on the spin systems considered and the parameters used are provided in Section S10 of the ESI.†

## Results and discussion

### ^17^O NMR spectroscopy: bulk structure of Al_2_O_3_

**Fig. 1:**
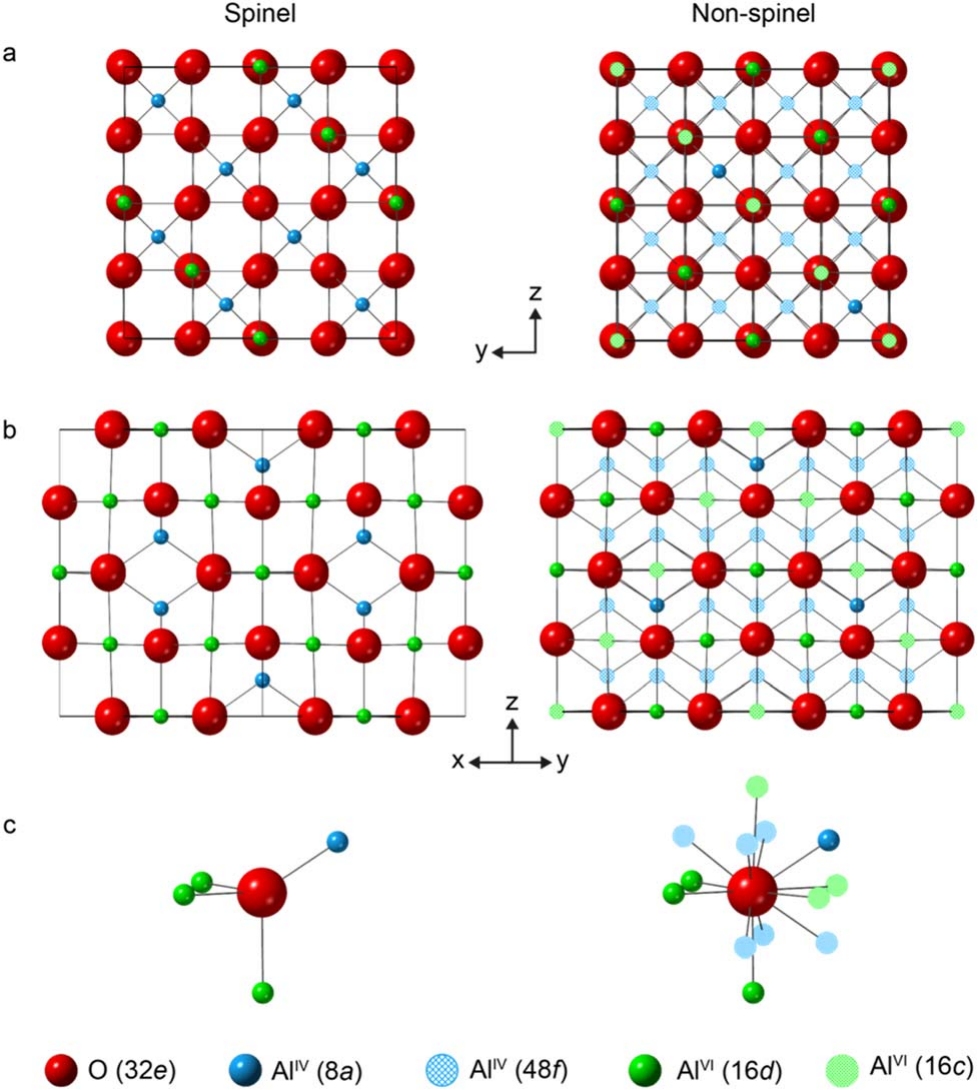
Schematic models for the structure of γ-Al_2_O_3_ based on a fcc arrangement of O^2−^ anions, and denoting the spinel and non-spinel cation positions, viewed (a) down the *x* axis and (b) down the (111) axis. (c) Local arrangement of the Al^3+^ cations around O in each of the models.

Fig. 1 shows structural models for γ-Al_2_O_3_ based on a cubic *Fd*3̄*m* unit cell containing an fcc arrangement of O^2−^ anions (on the 32e Wykoff positions), indicating both the spinel (8a and 16d) and non-spinel (48f and 16c) cation positions.^13^ In a spinel structure without cation vacancies, every O^2−^ anion is tetrahedrally coordinated and surrounded by one Al^IV^ and three Al^VI^ cations (as shown in Fig. 1c). To aid the interpretation of local structure in the following discussion, this O environment will be denoted as O^IV^(6664). To maintain charge balance in the spinel-based model of Al_2_O_3_, 8/3 Al^3+^ vacancies are needed for every 32 O^2−^ anions. Introducing an Al^VI^ vacancy generates six three-coordinate O species (denoted O^III^(664)), while an Al^IV^ vacancy generates four O^III^(666) sites. For non-spinel structures, there are 11 48f and 16c cations around each O^2−^ potentially giving rise to many different O local environments, as seen in Fig. 1c.

**Fig. 2:**
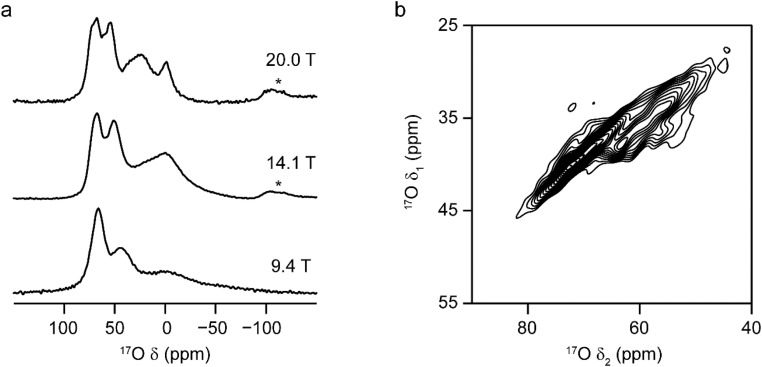
^17^O NMR of γ-Al_2_O_3_ enriched by post-synthetic exchange with ^17^O_2_ (g). (a) MAS NMR spectra of γ-Al_2_^17^O_3_(500 °C), acquired at the magnetic fields shown. (b) ^17^O (20.0 T, 30 kHz) DQF-STMAS^49^ NMR spectrum of γ-Al_2_^17^O_3_(950 °C). For detailed experimental parameters see Section S1 of the ESI.†

Fig. 2a shows ^17^O MAS NMR spectra of γ-Al_2_^17^O_3_ (500 °C), enriched for 48 h and then left open to the atmosphere, at three different *B*_0_ field strengths. The increase in field strength (and the consequent reduction in the second-order quadrupolar broadening) increases the spectral resolution and enables approximately four oxygen signals to be distinguished. The two sharper resonances at higher shift have been suggested to correspond to non-protonated oxygen species, while the two broader signals at lower shift have been previously assigned to protonated oxygens (*i.e.*, adsorbed water and hydroxyl groups).^42–45^ The signals from the non-protonated oxygens do not become significantly sharper at higher field, suggesting that the major contribution to the line broadening is a distribution of chemical shifts as a result of small variations in the local environments, rather than from a significant second-order quadrupolar interaction. Early ^17^O NMR of γ-Al_2_O_3_ by Walter and Oldfield^42^ assigned the non-protonated ^17^O signal at higher shift (∼70 ppm at 20.0 T) to O^IV^ and that at ∼55 ppm (at 20.0 T) to O^III^. Although this assignment is generally accepted in the literature,^43–46^ it will be shown later that this is likely to be an oversimplification. However, the “O^IV^” : “O^III^” ratio (1 : 1) obtained from ^17^O spectra in early work was later used^46^ to conclude that the cation vacancies in a spinel structure must be located exclusively at the octahedral positions (if they were at the tetrahedral sites, a 2 : 1 ratio would be obtained). This ratio was obtained by fitting the spectrum using Gaussian lineshapes, *i.e.*, without considering the characteristic “tails” to low shift usually seen for quadrupolar nuclei in disordered environments,^39^ and neglecting any effects of differential relaxation rates or differing nutation rates (*i.e.*, spectra were not acquired using a short flip angle pulse). Although the spectra in Fig. 2a in this work were acquired using sufficiently long recycle intervals (see Section S1 of the ESI†), it should be noted that the material was isotopically enriched by post-synthetic exchange with ^17^O_2_ (g), leading to possible non-quantitative incorporation of ^17^O. Although we would expect any differences to be insignificant, particularly when higher temperatures are used, any detailed structural conclusions in this work should not be based on spectral signal intensities alone but from the range and combination of experimental and computational evidence provided.

**Fig. 3:**
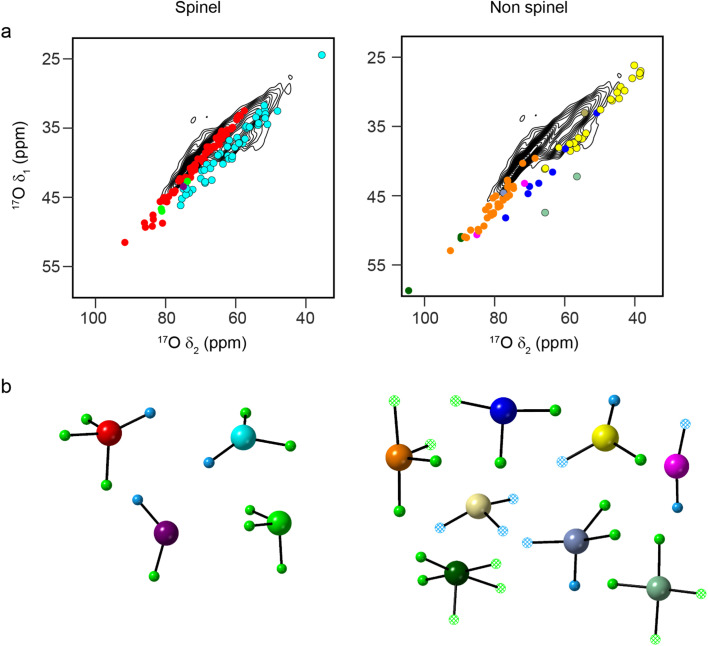
(a) Plots showing the overlay of (*δ*_1_, *δ*_2_) centre-of-gravity shifts predicted (using DFT) for different O environments in 3 × 1 × 1 supercells of cubic and tetragonal non-spinel structural models of γ-Al_2_O_3_ from Paglia,^61^ with the ^17^O (20.0 T, 30 kHz) DQF-STMAS spectrum of γ-Al_2_^17^O_3_(950 °C) from Fig. 2b. The colours denote the local oxygen environments present (shown in (b)), which have been separated into “spinel” and “non-spinel”, as defined in the text. See the ESI† for the full structural models.

Fig. 2b shows the ^17^O STMAS spectrum of γ-Al_2_O_3_(950 °C), a sample enriched by heating in a 70% ^17^O_2_ atmosphere at 800 °C for 6 h and then increasing the temperature to 950 °C before heating for further 14 h (Fig. S2.1 in the ESI† shows a direct comparison of the ^17^O MAS NMR spectra of γ-Al_2_O_3_ samples enriched at the two temperatures). The higher temperature leads to more efficient and more uniform enrichment (and therefore higher sensitivity) but results in small impurities of δ-Al_2_O_3_ and θ-Al_2_O_3_,^9,10^ as discussed in more detail in Section S2 of the ESI.† The spectrum shows two signals from the non-protonated O species – one centred at *δ*_1_ of ∼35 ppm and a second centred at higher *δ*_1_ (∼37. 5 ppm). Note that the signals from the protonated O species are not seen in the STMAS spectrum owing to very rapid relaxation (*vide infra*). To explore the likelihood of Al^3+^ cations occupying spinel or non-spinel environments, DFT calculations were used to predict ^17^O NMR parameters for two non-spinel structural models proposed by Paglia (using 3 × 1 × 1 supercells of the cubic and tetragonal unit cells).^61^ Schematics of the structural models used are shown in Section S3 of the ESI.† As shown in Fig. 3a, the oxygen sites present can be divided into two types based on their first coordination sphere: “spinel” sites (*i.e.*, local environments that would occur in structures where all cations are restricted to spinel positions) and “non-spinel” sites (*i.e.*, local environments that can occur when this restriction is not in place). Fig. 3b overlays the predicted positions (*δ*_1_, *δ*_2_) of the centre-of-gravity of the signal from each O species on the experimental ^17^O STMAS spectrum of γ-Al_2_^17^O_3_(950 °C) from Fig. 2b. The vast majority of the signals predicted for “non-spinel” species fall outside of the regions where signal is observed experimentally, while the majority of the signals predicted for the “spinel” species are in much better agreement with the experiment, indicating that γ-Al_2_^17^O_3_(950 °C) is unlikely to contain a high fraction of cations in non-spinel positions. It can also be seen that the signals from O^III^(644), shown in bright yellow (and also present in the low level δ-Al_2_O_3_ and θ-Al_2_O_3_ impurities^9,10^), overlay well with the shoulder seen on the STMAS lineshape, suggesting that this part of the signal may have a contribution from these impurities, as discussed in the ESI.† A second non-spinel model that is widely used for computational studies (as it contains only 14 atoms in the primitive unit cell) is that of Digne *et al.*,^62^ which has three types of O environment (O^IV^(6664), O^IV^(6666), and O^III^(664), shown in Fig. 3 in red, orange and cyan, respectively).

**Fig. 4:**
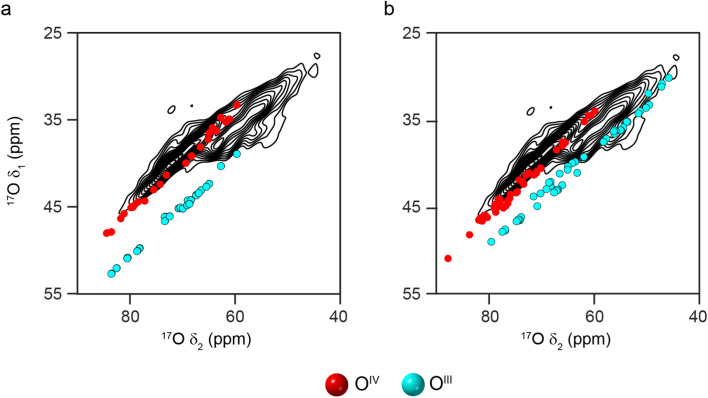
Plots showing the overlay of (*δ*_1_, *δ*_2_) centre-of-gravity shifts predicted (using DFT) for different O environments in 3 × 1 × 1 supercells of cubic spinel structural models of γ-Al_2_O_3_, with the ^17^O (20.0 T, 30 kHz) DQF-STMAS spectrum of γ-Al_2_^17^O_3_(950 °C) from Fig. 2b. In (a), vacancies appear only on Al^VI^ sites with maximum separation between them (see Model SP1 in the ESI†), while in (b), the distance between the vacancies is allowed to vary (see Model SP2 in the ESI†).

Given the poor agreement with ^17^O NMR experiments for O species that can only occur in non-spinel models of γ-Al_2_O_3_, only spinel models have been considered from this point forwards. Most previous computational studies on spinel-based models of γ-Al_2_O_3_ have used the smallest unit cell that contains an integer number of vacancies (*i.e.*, a 3 × 1 × 1 supercell of the 14 atom primitive cell with two cation vacancies).^11,14–20^ These suggested that lower energy structures were obtained when the cation vacancies were on the Al^VI^ sites and had the greatest separation between them. Here, we have used a larger model (a 3 × 1 × 1 supercell of the cubic spinel cell, with 160 atoms and eight cation vacancies). See the ESI† for more detail. Assuming the two requirements suggested above are retained, this model (Model SP1 in the ESI†) contains two types of O species; O^IV^(6664) and O^III^(664). As shown in Fig. 4a, the DFT-predicted position of the ^17^O STMAS signals do not agree well with the experimental data. Although the points corresponding to O^IV^ (red) match reasonably well with the ridge centred at lower *δ*_1_, the O^III^ (cyan) points match poorly with the second ridge of signal (appearing at much higher (*δ*_1_, *δ*_2_) than seen in experiment), suggesting that at least one of the assumptions stated above is incorrect. If Al^VI^ vacancies are retained, but the requirement for maximum separation between them is not strictly enforced (see Model SP2 in the ESI†), the O^III^ signals predicted by DFT (see Fig. 4b) cover a much wider spectral range and overlap with some of the signal seen experimentally. The O^III^(664) sites with lower *δ*_iso_ values correspond to O species which are next to one Al^VI^ vacancy but are also close to a second. For O sites with *δ*_2_ between 44 and 58 ppm, (corresponding to *δ*_iso_ between 50-64 ppm) the next nearest vacancy is ∼3.4 Å away, while for those with *δ*_2_ > 62 ppm (corresponding to *δ*_iso_ > 68 ppm), this distance is ∼4.3 Å. This suggests that γ-Al_2_^17^O_3_(950 °C) exhibits preferential clustering of the cation vacancies, contrary to the conclusions of previous computational studies.^11,14–20^ However, the O sites present in the models used for the DFT calculations have slightly larger *P*_Q_ values than those seen experimentally (and are more similar to the O^III^(664) sites in δ-Al_2_O_3_ and θ-Al_2_O_3_).

**Fig. 5:**
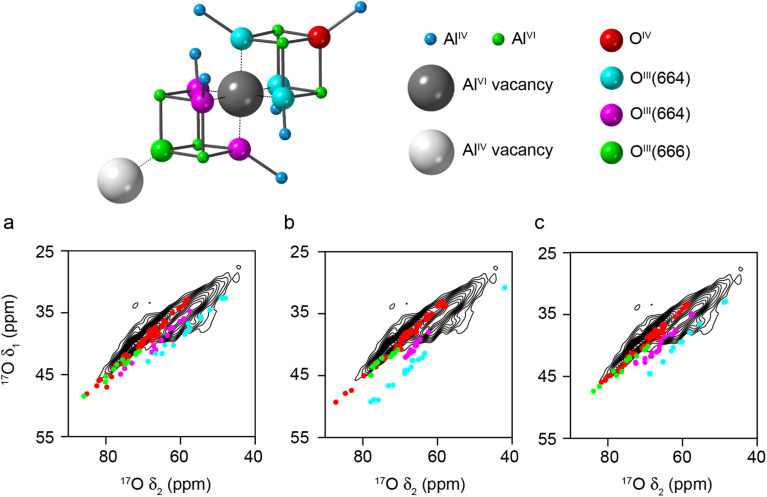
Plots showing the overlay of (*δ*_1_, *δ*_2_) centre-of-gravity shifts predicted (using DFT) for different O environments in three different structural models (Models SP3-5, for (a), (b) and (c), respectively) generated from 3 × 1 × 1 supercells of cubic spinel structural models of γ-Al_2_O_3_, with the ^17^O (20.0 T, 30 kHz) DQF-STMAS spectrum of γ-Al_2_^17^O_3_(950 °C) from Fig. 2b. In each structure, vacancies are present on Al^VI^ and Al^IV^ sites with maximum separation between them (see Fig. S3.4 of the ESI†). The key above shows the atom types and vacancies present.

If the requirement for only Al^VI^ vacancies in the spinel model is also relaxed, and Al^IV^ vacancies are introduced, different O environments are then observed, as shown in Fig. 5 (see Models SP3-5 in the ESI†). Fig. 5a–c show the DFT-predicted position of the ^17^O STMAS signals for the O species in three such models. The signals seen for a new O species, O^III^(666), shown in green, are very similar to the red points corresponding to O^IV^(6664), and in the ^17^O MAS spectrum would primarily contribute to the O signal at the highest *δ* (previously assumed by Walter and Oldfield^42^ to result only from O^IV^). It is also clear that O^III^(664) signals fall into two distinct groups; those shown in cyan are very similar to those seen previously in Fig. 4 for structures where only Al^VI^ vacancies are present, and those shown in magenta, which have a smaller *P*_Q_ value (∼3 MHz compared to ∼4 MHz) and are much closer to the values seen experimentally, matching well with the second ridge centred at higher *δ*_1_. The difference between these two chemically similar species can be seen in Fig. 5, which shows that the second type of O^III^(664) (magenta) is next to an Al^VI^ vacancy, and the next nearest Al^IV^ along the (110) planes is also missing. However, for the O^III^(664) (cyan) this Al^IV^ is present. The O^III^(664) magenta sites match much better with experimental measurements than the O^III^(664) cyan sites, suggesting that it is likely that alternating Al^VI^ and Al^IV^ vacancies are present in γ-Al_2_^17^O_3_(950 °C), and that there are roughly equal numbers of these.

Structural models with alternating Al^VI^ and Al^IV^ vacancies can be generated (see Models SP6-8 in the ESI†). However, by definition, these must contain a line of Al^IV^–Al^VI^–Al^IV^ vacancies (see Fig. 5), although the next Al^VI^ vacancy can then be in the direction of any of the three neighbouring O atoms. In a real crystal this would generate complex vacancy “trajectories” and lead to many different O species in the resulting disordered solid. However, when limited to a 160 atom supercell this results in only a small number of possibilities and ordered structural models, leading to a relatively small number of distinct O species, which do not represent well the range that would appear in reality. The DFT-predicted ^17^O STMAS signals for these more ordered models are shown in Fig. S3.5 of the ESI.† The DFT calculations reproduce the experimental *P*_Q_ values well, but the *δ*_iso_ values show a poorer match to experiment for the O^III^(664) sites shown in magenta in Fig. 5, owing to the limited cell size and the ordered structures that result. In these models the O^III^(664) species are either very close to, or very far from, a third Al vacancy (giving rise to very low or very high *δ*_iso_ values, respectively). In a real material, a range of distances would be expected owing to the more disordered longer-range structure, leading to intermediate *δ*_iso_ values in better agreement with experiment; however, this would require much more computationally costly calculations to explore in detail.

Although it is well known that the surface of γ-Al_2_O_3_ is hydroxylated and hygroscopic, some studies have also suggested the presence of H in the bulk structure (from the boehmite precursor most commonly used in synthesis).^63,64^ The only NMR spectroscopic study that supports this proposition was carried out a number of years ago using continuous wave (CW) NMR of static samples, and concluded that 37% of all protons are located in the bulk γ-Al_2_O_3_.^65^ The more recent work of Paglia^66^ did not report any evidence for significant levels of bulk H, a conclusion which is supported further by the fact that γ-Al_2_O_3_ can also be produced from non-hydrogenous precursors.^67^ To investigate the possibility of significant levels of ^1^H in the bulk structure of γ-Al_2_O_3_, DFT calculations were carried out for four further structural models (Models SPH1-4), generated as described in Section S4 of the ESI† by replacing one Al^3+^ cation with three H^+^. The DFT-predicted positions of the ^17^O STMAS signals are shown in Fig. S4.1 of the ESI.† The substitutions generate a new type of O species that acts as a hydrogen bond acceptor (H–O^III^(664)), which have similar NMR parameters to their non-hydrogen-bonded counterparts. No experimental ^17^O STMAS signals are seen for the protonated surface O species, owing to the rapid relaxation that results from dynamics between aluminols (*i.e.*, Al–OH species) and exchange with water (see later). However, such dynamics are likely to be much less pronounced for H in the bulk, and the corresponding protonated O species may be expected to appear in the ^17^O STMAS spectrum. Fig. S4.1† shows no evidence for these signals (also predicted to be at much lower *δ*) experimentally. There is also little evidence for the presence of bulk H in the experimental ^1^H DEPTH MAS NMR spectrum of dehydrated γ-Al_2_^17^O_3_(550 °C) shown in Fig. S4.2 of the ESI,† with no significant signal seen at the DFT-predicted *δ*_iso_ values. In general, therefore, there is no evidence that γ-Al_2_O_3_ contains a significant amount of H in the bulk structure.

Although it is generally accepted that the O^2−^ anions form an fcc array, the presence of stacking faults has been reported^68^ and the presence of oxygen vacancies also proposed.^69,70^ To investigate the latter, two further models of γ-Al_2_O_3_ were considered, with the removal of 3 O^2−^ and 2 Al^3+^ cations from Model SP6. In Model SPV1 (see Section S5 of the ESI†) the O vacancies are more remote in the structure, whereas in Model SPV2 an “Al_2_O_3_ unit” was removed with all of the vacancies clustered. In both cases, many new types of oxygen environments are created that exhibit NMR parameters in poor agreement with the signals in ^17^O STMAS spectra (see Fig. S5.1 in the ESI†). This suggests that if such vacancies are present in bulk γ-Al_2_O_3_, they most likely occur only at very low levels.

The results discussed so far present an overall picture of bulk γ-Al_2_O_3_ as having a fcc array of oxide ions with Al^3+^ occupying only spinel sites, with clusters and/or chains of Al vacancies on both Al^IV^ and Al^VI^ sites. The bulk appears to be anhydrous and there is little evidence of any significant disruption to the fcc array of O atoms (either stacking faults or clustered vacancies). While it is clear that O^IV^ and O^III^ species are present, our computational work suggests that these signals may overlap more than had been previously suggested (and the assignments used in previous work may be a simplification).

### ^17^O NMR spectroscopy: surface structure of Al_2_O_3_

Although there has been considerable debate over the structure of bulk γ-Al_2_O_3_, an understanding of the surface structure is more relevant from a catalysis perspective, and has been the focus of much previous computational work.^5,15,19,21,62,64^ The three surface planes of γ-Al_2_O_3_, (100), (110) and (111) as indexed in the cubic spinel unit cell,^5^ are shown in Section S6 of the ESI,† where the surface arrangement of the O^2−^ anions and Al^3+^ cations are also highlighted. Unlike the (100) and (100) planes, normal to the (111) planes there are alternating layers of O^2−^ and Al^3+^ ions. Considering cation vacancies leads to a significantly larger number of inequivalent cleavages that could be performed. Structural models of “γ-Al_2_O_3_ slabs” for DFT calculations were generated from the bulk models of γ-Al_2_O_3_ by cleaving along the three surface planes as described in the ESI,† giving rise to 36 structural models (22 fully and 14 partially hydroxylated structures, each with two surfaces).

**Fig. 6:**
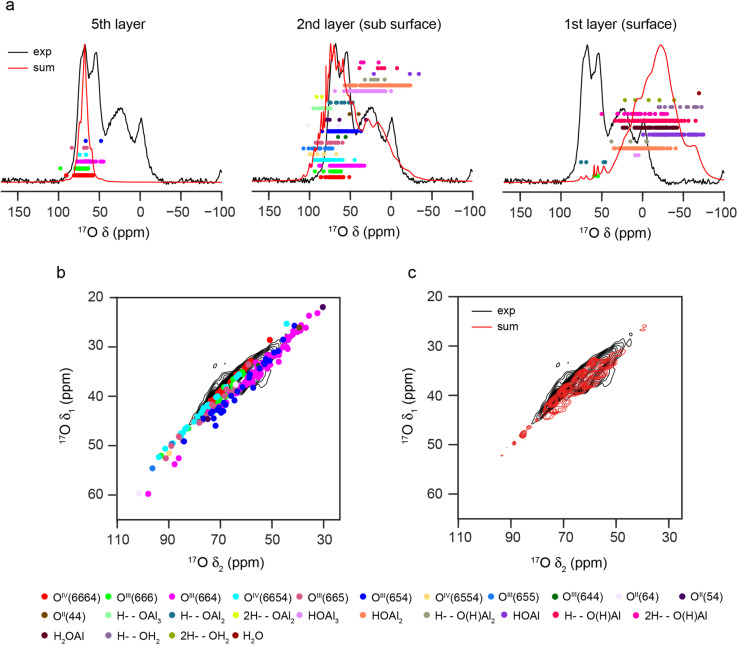
(a) Plots showing the overlay of the DFT-predicted NMR shifts (*δ*_2_) for all O in the slab models of γ-Al_2_O_3_, separated by the number of layers from the surface, with the ^17^O (20.0 T, 20 kHz) MAS NMR spectrum of γ-Al_2_^17^O_3_(500 °C) from Fig. 2a. The red line results from co-adding Lorentzian lineshapes simulated for each O species, centred at the *δ*_2_ centre-of-gravity with a FWHH proportional to *C*_Q_. (b and c) Plots showing the overlay of (*δ*_1_, *δ*_2_) centre-of-gravity shifts predicted (using DFT) for non-protonated O environments in sub surface layer of the slab models of fully hydroxylated γ-Al_2_O_3_, with the ^17^O (20.0 T, 30 kHz) DQF-STMAS spectrum of γ-Al_2_^17^O_3_(950 °C) from Fig. 2b. In (c), the red contours represent the spectrum simulated from the sum of Lorentzians for each of the points in (b), with a FWHH of 1.5 ppm in *δ*_1_ and *C*_Q_/MHz ppm in *δ*_2_ the ESI.†

Fig. 6a overlays the ^17^O DFT-calculated *δ*_2_ centre-of-gravity shifts (*i.e.*, including both *δ*_iso_ and any second-order quadrupolar shift) on the experimental ^17^O MAS NMR spectrum of γ-Al_2_^17^O_3_(500 °C). This is shown separately for oxygens in the surface layer, the sub surface layer and five layers from the surface. See Fig. S6.3 in the ESI† for the corresponding plots for O in layers three and four from the surface, and for plots of the (*δ*_1_, *δ*_2_) centre-of-gravity shifts for the non-protonated O in each of the five layers shown overlaid on the ^17^O STMAS spectrum of γ-Al_2_^17^O_3_(950 °C). For O species that are four and five oxide layers from the surface (not including any water layer), the NMR parameters are very similar to those calculated for O in the bulk models of γ-Al_2_O_3_ discussed above, although the breaking of symmetry by the surface cleavage gives rise to a larger distribution of NMR parameters. The predicted shifts agree well with those seen experimentally. However, three layers from the surface (see Fig. S6.3 in the ESI†) a much greater number of different types of non-protonated O species are seen (owing to the different coordination numbers of the neighbouring Al cations, many of which are now reduced by the cleavage). Three layers from the surface there is also an increasing number of O with lower *δ*_iso_ (and *δ*_2_) shifts, providing insight into the observation above that the most downfield resonance in the ^17^O MAS NMR spectrum increases in intensity when higher enrichment temperatures are used, enabling diffusion of ^17^O deeper into the material. In the sub surface layer, there is a large variety of protonated and non-protonated O environments present, which span the experimentally observed shift range. However, there are several points that fall outside of this range, notably for O^IV^(6554) and O^III^(655). These environments primarily occur in partially hydroxylated structures and so are not generally likely to be observed experimentally, where surfaces are fully hydroxylated and hydrated. Fig. 6b shows the overlay of the DFT-predicted (*δ*_1_, *δ*_2_) centre-of-gravity shifts for non-protonated O environments in the sub surface layers of the slab models of γ-Al_2_O_3_, with the ^17^O (20.0 T, 30 kHz) DQF-STMAS spectrum of γ-Al_2_^17^O_3_(950 °C) from Fig. 2b. This shows relatively few points outside of the region where signals are observed experimentally, an observation which is perhaps illustrated more clearly in Fig. 6c, where Lorentzian lineshapes are applied to each of the points in Fig. 6b and summed. This result should not be interpreted as an indication that γ-Al_2_^17^O_3_(950 °C) is enriched in ^17^O only as far as the sub surface layer but suggests that the variations in local geometry that arise from the presence of surfaces cannot be modelled well in the bulk models of γ-Al_2_O_3_ used above.

**Fig. 7:**
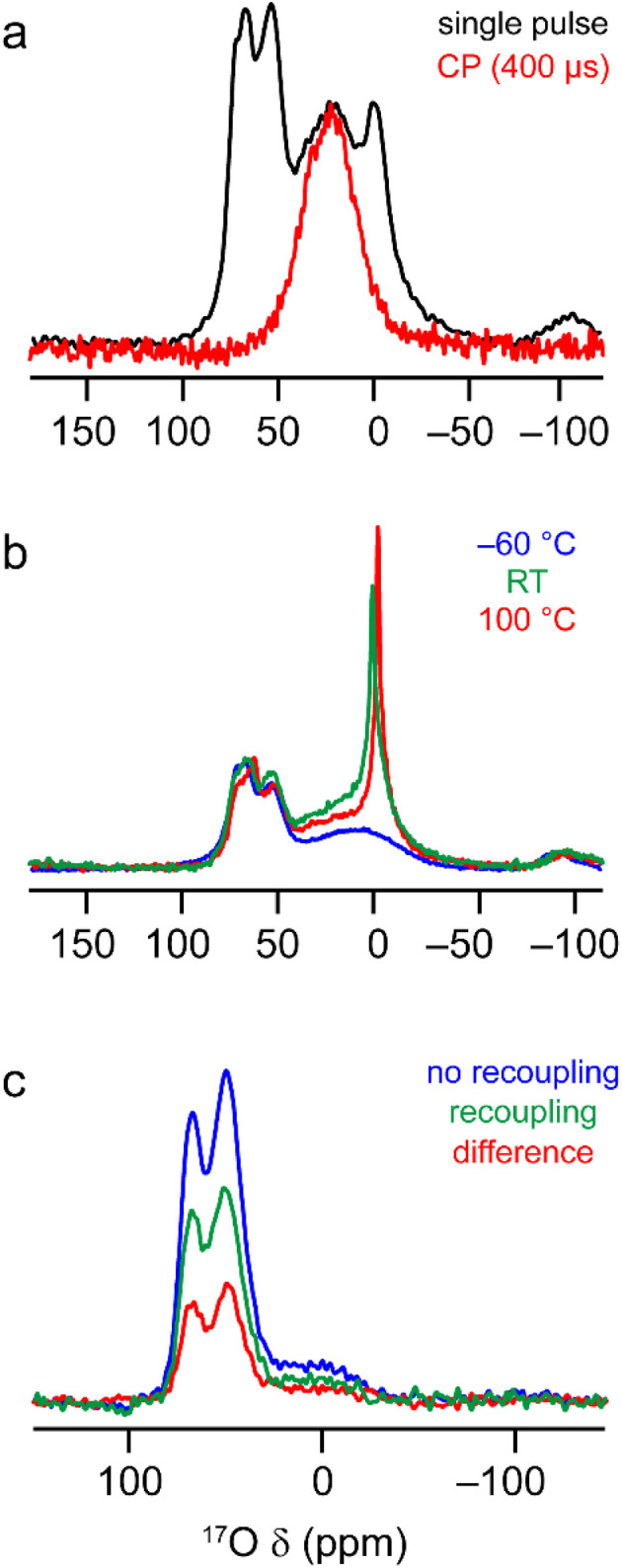
^17^O MAS NMR of γ-Al_2_^17^O_3_(500 °C). (a) (20.0 T, 20 kHz) MAS and CP MAS NMR spectra acquired with *τ*_CP_ = 400 μs. (b) (20.0 T, 20 kHz) MAS NMR spectra acquired at varying temperatures. (c) (14. 1 T, 14 kHz) ^17^O–^27^Al TRAPDOR spectra acquired with *τ*_rec_ = 16 *τ*_R_. See Section S7 of the ESI† for more detailed experimental parameters and information on normalisation and scaling.

The ^17^O MAS NMR spectrum of γ-Al_2_^17^O_3_(500 °C) acquired at 20.0 T, shown in Fig. 7a, contains two signals from primarily protonated O species; a broad resonance at ∼20 ppm and a sharper signal at −2 ppm (see Fig. S7.1 in the ESI† for results at 14.1 T). These signals have very short relaxation times (*T*_1_ of ∼1.6 and ∼0.4 ms, respectively, as measured by saturation recovery experiments, and *T*_2_′ of ∼0.6 and ∼0.1 ms, measured using rotor-synchronised single spin-echo experiments). This contrasts with the relaxation seen for the signals at higher shifts from non-protonated O (*T*_1_ of ∼1–1.5 s and *T*_2_′ of ∼10 ms), reflecting the involvement of the protonated O species in dynamic surface processes and their interactions with water. From Fig. 6a, the surface layers of the γ-Al_2_O_3_ models contain only protonated O, with the majority of calculated *δ*_2_ values lower than the signals seen experimentally. However, the *δ*_2_ values for the protonated O in the sub surface layer match very well with the experimentally observed broader resonance, suggesting this results from less accessible O sites. The sharper resonance then likely results from averaging of the more accessible surface sites (which is also reflected in the faster *T*_1_ and *T*_2_ relaxation times for this signal). This is also supported by the observation that only the broader signal is seen in ^1^H–^17^O cross polarisation (CP) spectra, as shown in Fig. 7a. Further evidence to support the assignment of the sharp signal at −2 ppm to O in the surface layers is given in Fig. S7.2 in the ESI,† where it can be seen to vary in intensity with time and storage conditions. The ^17^O MAS NMR spectra of γ-Al_2_^17^O_3_(500 °C) acquired at different temperatures, and shown in Fig. 7b, reveal a significant change in linewidth of the sharp signal at lowest *δ*_2_, with broader lines seen at lower temperatures (suggesting dynamics are in the fast-intermediate regime). At −60 °C, the sharp resonance becomes overlapped with the broader signal at higher *δ*_2_. Very little change is seen in the corresponding CP MAS NMR spectra, which still contain only the broader signal.

The computational results suggest that the broad signal for protonated O (at ∼20 ppm at 20.0 T) in the ^17^O MAS NMR spectrum arises primarily from aluminols (Al–OH species). However, there is an argument that this signal could, alternatively, be assigned to less mobile or strongly hydrogen bonded H_2_^17^O molecules. These species are likely to be much further from Al^3+^ cations and so the two types of signal could potentially be distinguished using ^17^O-^27^Al dipolar dephasing measurements. Fig. 7c shows ^17^O-^27^Al TRAPDOR^71^ spectra of γ-Al_2_^17^O_3_(500 °C), which demonstrate the presence of the broad signal from protonated O in the dephasing difference spectrum and confirm its assignment to aluminols. See the ESI† for a more detailed discussion of the recoupling experiments.

### ^27^Al and ^1^H NMR spectroscopy of Al_2_O_3_

**Fig. 8:**
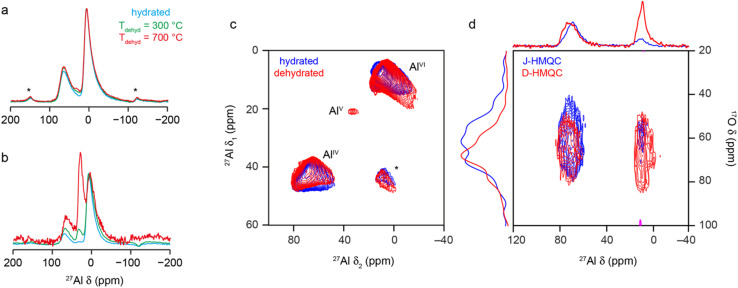
^27^Al (9.4 T, 14 kHz) (a) MAS and (b) CP MAS NMR spectra of γ-Al_2_O_3_, hydrated (cyan) and dehydrated at 300 °C (green) and 700 °C (red). (c) ^27^Al (14.1 T, 14 kHz) MQ MAS spectrum of γ-Al_2_O_3_ hydrated (blue) and dehydrated at 300 °C (red). (d) ^27^Al–^17^O (20.0 T, 20 kHz) J-HMQC (blue) and D-HMQC (red) spectra of γ-Al_2_^17^O_3_(950 °C). For D-HMQC, SR4^2^_1_ recoupling was applied for 0.9 ms. For detailed experimental parameters see Section S1 of the ESI.†

As discussed briefly above, many of the previous spectroscopic studies of γ-Al_2_O_3_ have focussed on ^27^Al NMR, which is sensitive to the Al coordination number (with Al^IV^, Al^V^ and Al^VI^ species found at *δ* = 80 to 50 ppm, 40 to 20 ppm and 20 to −20 ppm, respectively, although exact shifts for a material are field dependent as *I* = 5/2 for ^27^Al). The ^27^Al MAS NMR spectrum (acquired with a short flip angle) of γ-Al_2_O_3_ in Fig. 8a shows two broad signals, corresponding to Al^IV^ and Al^VI^, in a *ca.* 1 : 2 ratio, with both exhibiting the tails to low frequency characteristic of disordered materials.^39^ The ^27^Al MQMAS spectrum in Fig. 8c confirms distributions of both *δ*_iso_ and *P*_Q_ are present (with average values as given in Section S8 of the ESI†). ^27^Al–^17^O HMQC^72^ experiments were performed both without (*i.e.*, J-HMQC) and with (D-HMQC) dipolar recoupling (using SR4^2^_1_ ^73^). For D-HMQC (as shown in Fig. 8d), all O signals correlate with Al^IV^ and Al^VI^ species as expected. However, for J-HMQC experiments the correlation is much stronger with Al^IV^ than with Al^VI^. This somewhat surprising result may be understood by considering the values of the ^27^Al–^17^O *J* couplings for these two environments. These were calculated for Model SP6 of bulk γ-Al_2_O_3_, showing that Al^VI^ species have much smaller *J* couplings (*e.g.*, ∼3–10 Hz) than Al^IV^ (*e.g.*, ∼7–20 Hz), reflecting the higher ionicity (and consequently longer Al–O bonds) for Al^VI^. See Section S9 of the ESI† for more detail. This would require longer HMQC evolution periods for efficient magnetisation transfer for Al^VI^, although accessible values are ultimately limited by *T*_2_ (3–5 ms).

Al^V^ species are not easily observed in the MAS or MQMAS spectra of hydrated bulk γ-Al_2_O_3_, although their low-level presence has been demonstrated previously.^32,74–77^ Early studies^32^ showed the concentration of Al^V^ depends on the exact synthetic route and conditions used, and particularly on any grinding of the precursor prior to calcination. Most studies indicate Al^V^ sites are preferentially, if not exclusively, located at the surface. As discussed above, there is little evidence in the materials studied here for H in the bulk, and so ^1^H–^27^Al CP experiments can be used to selectively probe the alumina surface. For the hydrated γ-Al_2_O_3_, a low intensity Al^V^ signal can be seen between 20 and 40 ppm in the CP MAS spectrum in Fig. 8b. Dehydration will also affect the surface structure, increasing the relative intensity of Al^IV^ and Al^V^ species in the MAS and CP MAS NMR spectra (as shown in Fig. 8) with increasing dehydration temperature. Although not shown, the MAS spectrum does not change for samples dehydrated at 500 and 700 °C, suggesting the lower temperature is sufficient to remove the adsorbed water molecules. Dehydration yields a higher concentration of Al^V^ and their observation (albeit with very low intensity, at *δ*_1_ = 20 ppm) in the ^27^Al MQMAS spectrum (as shown in Fig. 8c), with average NMR parameters as shown in Table S8.1 the ESI.†

**Fig. 9:**
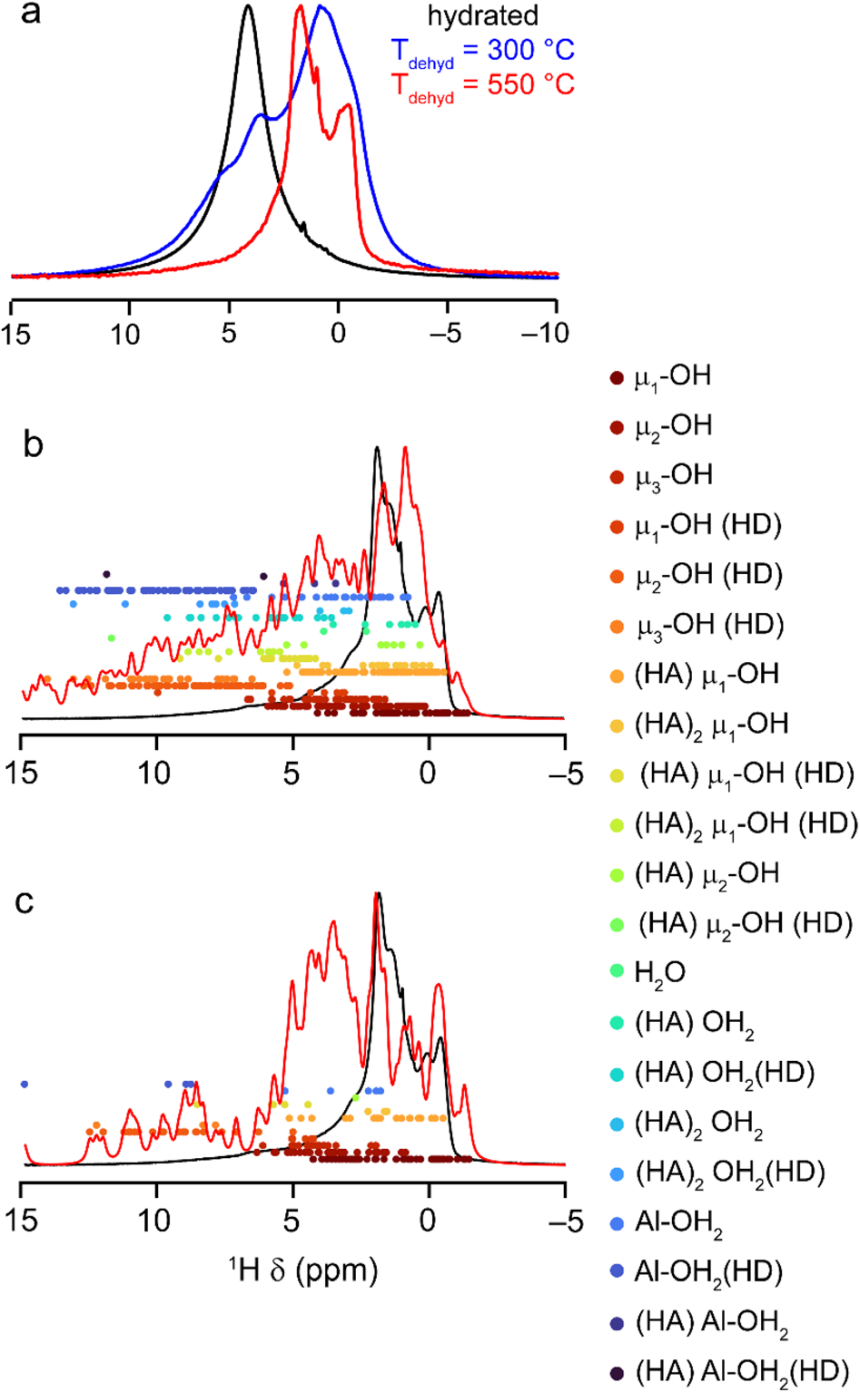
(a) ^1^H (9.4 T, 14 kHz) DEPTH MAS NMR spectra of γ-Al_2_O_3_ hydrated (black) and dehydrated at 300 °C (blue) and 550 °C (red). (b and c) Plots showing the overlay of the DFT-predicted NMR shifts (*δ*_iso_) for all H in the (b) fully and (c) partially hydroxylated slab models of γ-Al_2_O_3_, overlayed with the ^1^H (14.1 T, 20 kHz) DEPTH MAS NMR spectrum of γ-Al_2_O_3_ dehydrated at 550 °C. The red line represents the spectrum simulated from the sum of Lorentzians (FWHH of 150 Hz) added for each of the points shown. For detailed experimental parameters see the ESI.†

Early work using IR spectroscopy^78^ suggested the presence of at least 5 different types of OH groups at the surface of γ-Al_2_O_3_. However, the ^1^H MAS NMR spectrum of hydrated γ-Al_2_O_3_ is dominated by a broad resonance from water (∼4.5 ppm) that obscures signals from surface aluminol groups, as shown in Fig. 9a. Upon dehydration, more complex spectral lineshapes are revealed. After dehydration at 300 °C, the lineshape is still relatively broad, but two distinct components (at ∼3.7 and 0.7 ppm) are resolved, with a shoulder at ∼5.1 ppm. When dehydration is carried out at a higher temperature (550 °C) a larger number of sharper signals are observed (with most between 0 and 4 ppm), although these are still significantly overlapped. The slow rehydration of the surface inside the NMR rotor (leading to increasing intensity at higher shifts) is shown in Fig. S10.1 in the ESI.† It should be noted that no significant decrease in linewidth was observed with increasing MAS rate or with the application of homonuclear decoupling, suggesting that the linewidth results primarily from a distribution of chemical shifts.

The ranges of ^1^H *δ*_iso_ predicted using DFT for the 22 fully and 14 partially hydroxylated models of γ-Al_2_O_3_ described above are shown in Fig. 9b and c, respectively. The ^1^H environments are grouped according to the denticity of the hydroxyl groups (with μ_1_ being non bridging or terminal, μ_2_ bridging and μ_3_ a triply-bridged OH group), the coordination number of the Al^3+^ co-ordinated to the O, and whether the H is a hydrogen bond donor (HD) or the O a hydrogen bond acceptor (HA). The OH distance was considered a covalent bond when <1.2 Å. The spectra obtained from summing Lorentzians (FWHH of 150 Hz) of unit intensity applied to each data point do not match well with the experimental spectra in either case, but the range of shifts is reproduced well, and the spectra help in understanding the changes to the ^1^H MAS NMR spectrum seen with hydration. For a group of μ_*n*_^1^H, those H that act as hydrogen bond donors have higher shifts in each case. It is also noticeable that signals between 0 and 1 ppm result primarily from μ_1_ and μ_2_ hydrogen bond acceptor aluminols), in good agreement with the previous work of Deng and co-workers.^43^ Similar plots considering predicted shifts for μ_1_, μ_2_ and μ_3_ separately are given Fig. S10.2 in the ESI† (where the coordination numbers of the coordinated Al^3+^ are also denoted in each case). Comparison of these plots confirms that the average ^1^H shift increases with denticity, negative shifts are only observed for non-bridging aluminols and that there is little dependence of the ^1^H shift on the coordination number of the Al^3+^ around O.

**Fig. 10:**
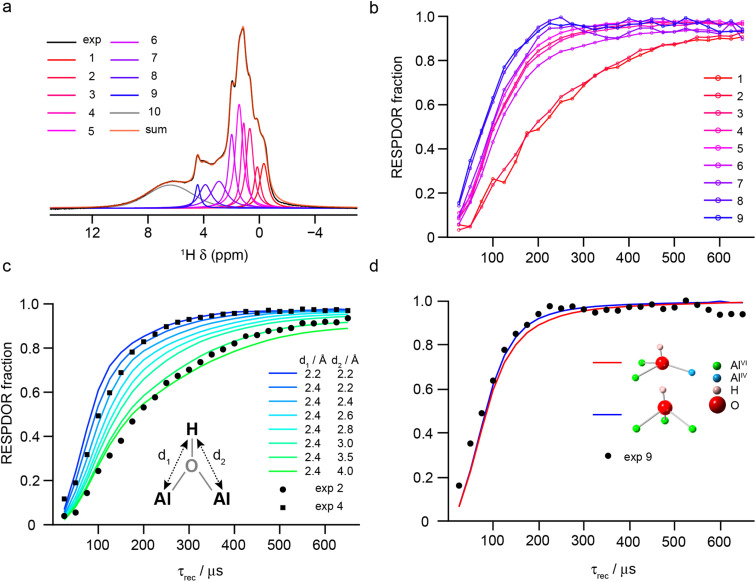
(a) Decomposition of the ^1^H (14.1 T, 40 kHz) DEPTH MAS NMR spectrum of γ-Al_2_O_3_ dehydrated at 550 °C (blue) into the 10 components used in fits of the RESPDOR spectra. (b) Experimental RESPDOR curves (acquired using R12_3_^5^ recoupling) plotted as a function of *τ*_rec_. (c) Overlay of experimental RESPDOR curves for components 2 and 4 with corresponding plots from numerical simulations of a three-spin (H_2_Al) system with varying H–Al distances, as shown. (d) Overlay of the experimental RESPDOR curve for component 9 with corresponding plots from numerical simulations of two four-spin (H_3_Al) systems for μ_3_ aluminols extracted from the structural models above, as shown. In (c) and (d), the ^27^Al *C*_Q_ was 5 MHz and in (c) the angle between the internuclear vectors was also fixed at 60°. For detailed experimental parameters see Section S1 of the ESI.†

The denticity of the aluminol groups that produce different signals in the ^1^H MAS NMR spectrum was investigated experimentally using RESPDOR^79^ to measure ^1^H–^27^Al dipolar couplings (see Section S1 of the ESI† for the experimental details). The ^1^H MAS NMR spectrum was decomposed into 10 separate Voigt components, nine of which were then used for analysis (see Section S10 of the ESI†), as shown in Fig. 10a. Fig. 10b shows the nine RESPDOR fractions ((*S*_0_ – *S*)/*S*_0_) as a function of *τ*_rec_. Three groups of signals can be clearly distinguished, corresponding to components 1–2, 3–7 and 8–9. In Fig. 10c, the RESPDOR curves for components 2 and 4 are compared with numerical simulations for a ^1^H(^27^Al)_2_ spin system, with distances *d*_1_ and *d*_2_ determining the two different heteronuclear dipolar couplings. For all simulations, the ^27^Al *C*_Q_ = 5 MHz and the angle between the two internuclear vectors was fixed at 60°. Variation in these parameters has only a small effect on the RESPDOR fraction calculated (as shown in the ESI†). For component 2, a good match is obtained when *d*_1_ < *d*_2_ (*i.e.*, a μ_1_ aluminol), which agrees with the DFT-predicted shift of these species. For component 4, simulation shows the best match is for *d*_1_ = *d*_2_ ≈ 2.4 Å, suggesting this group of signals result from μ_2_ aluminols, which make up the most abundant H environments on the γ-Al_2_O_3_ surface. Finally, as shown in Fig. 10d RESPDOR curves were simulated for the two most common types of μ_3_ aluminols (spin system geometries were directly extracted from the structural models described above, with *d*_1_, *d*_2_ and *d*_3_ set to 2.4 Å). The simulation is in good agreement with the experimental result, confirming that components 8–9 (*δ*_iso_ = 4–5 ppm) can be attributed primarily to triply-bridging OH groups. These results agree with those of Taoufik *et al.*,^77^ who were able to identify μ_1_ and μ_2_ aluminols, but also provides the assignment of signals from μ_3_ aluminols, which have previously only been tentatively assigned based on their predicted higher shifts.

**Fig. 11:**
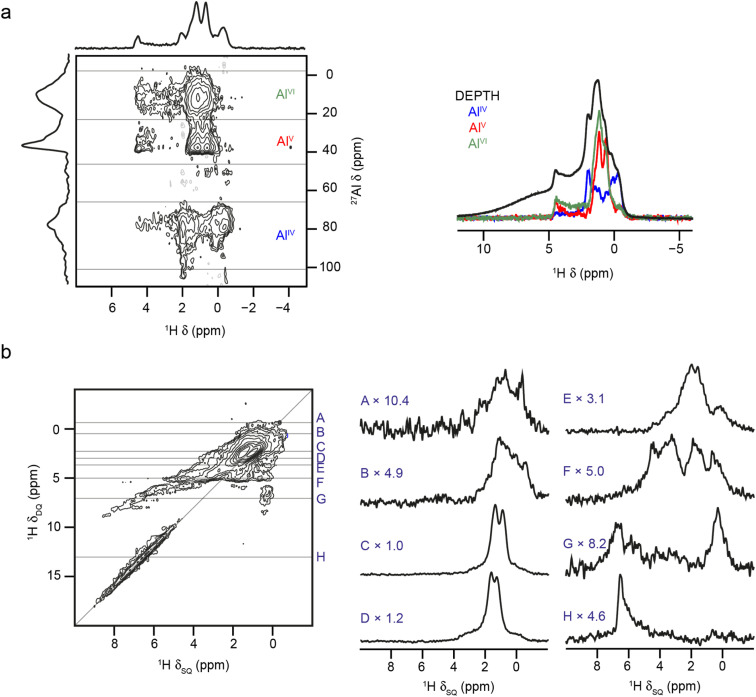
(a) ^1^H–^27^Al (14.1 T, 40 kHz) D-HMQC spectrum (with TRAPDOR recoupling) of γ-Al_2_O_3_ dehydrated at 550 °C, with sum projections (extracted parallel to *δ*_2_) over the regions corresponding to Al^IV^, Al^V^ and Al^VI^ (as shown), overlaid with the ^1^H DEPTH (14.1 T, 40 kHz) DEPTH MAS NMR spectrum of γ-Al_2_O_3_ dehydrated at 550 °C. (b) ^1^H (14.1 T, 40 kHz) DQ MAS spectrum (acquired using BABA recoupling) of γ-Al_2_O_3_ dehydrated at 550 °C, with cross sections (extracted parallel to *δ*_2_ and vertically expanded as shown) extracted at the *δ*_1_ positions indicated. For detailed experimental parameters see Section S1 of the ESI.†

The coordination numbers of the Al cations attached to the aluminols were determined using ^1^H–^27^Al D-HMQC experiments (with TRAPDOR recoupling), as shown in Fig. 11a. Sum projections for shift regions corresponding to Al^IV^, Al^V^ and Al^VI^ show that μ_1_ aluminols exhibit a weaker correlation with Al^V/VI^ than with Al^IV^ (a result consistent with previous work by Taoufik *et al.*^77^ and Szeto *et al.*^80^). This reflects not only the higher abundance of Al^IV^ on partially dehydroxylated surfaces, but the increased difficulty of removing OH groups from these species (as opposed to Al^V^ and Al^VI^) species, as this would generate metastable Al^III^ environments. The existence of Al^III^ sites on alumina surfaces has been hypothesised in a range of previous work considering the reaction of N_2_, H_2_, CH_4_, pyridine, CO and CO_2_ with alumina, and studied using computational approaches, NMR spectroscopy and IR spectroscopy.^81–85^ The Al^III^ species are thought to result from high-temperature pre-treatment of the surface (typically at ∼700 °C) and to be largely responsible for the adsorption of N_2_ or pyridine and the splitting of CH_4_ and H_2_.^82–85^ However, it has also been shown that the level of water present plays an important role both in the changing the reactivity seen and in varying the coordination number of the Al species at the surface.^81–83^ Direct detection of Al^III^ species (if present) using NMR spectroscopy would be extremely challenging, owing the large quadrupolar couplings expected. In the current work, for μ_2_ aluminols, coordination to all three types of Al is observed, but at higher *δ*_H_ the correlation to Al^V^ and Al^VI^ is weaker, suggesting high levels of μ_2_-OH (Al-44), while the reverse is true at lower *δ*_H_, suggesting these ^1^H signals result from species such as μ_2_-OH (Al-56) and μ_2_-OH (Al-66). For ^1^H signals corresponding to μ_3_ aluminols, correlations to Al^IV^ are weaker, suggesting the most abundant species are μ_3_-OH (Al-666), μ_3_-OH (Al-665), *etc.* The spatial distribution of aluminols can be investigated using ^1^H DQ MAS spectroscopy,^86^ as in previous work.^77^Fig. 11b shows that γ-Al_2_O_3_ dehydrated at 550 °C correlations between most types of aluminol ^1^H are observed, although with differing intensities. This suggests no sites are exclusively isolated, but that the closest proximities are between bridging aluminols coordinated to Al^IV^ and/or Al^V^ species (traces C and D). This is perhaps not surprising given these are the most abundant species present on the surface, and these species also show correlations with all other types of ^1^H (traces A, B, E and F). This observation can also be understood by considering the topology of the (100) and (110) surfaces (as shown in Fig. S6.1†). The (100) surface contains chains of Al^V^ species which can, upon hydroxylation, accommodate neighbouring μ_2_-OH (Al-66) environments that do not share Al^3+^ cations. At the (110) surface, chains of Al^IV^ sites (generated from Al^VI^) are present, which can give rise to continuous chains of μ_2_-OH (Al-66) sharing Al^VI^ cations, upon hydroxylation. This can also give rise to μ_2_-OH (Al-56) and μ_2_-OH (Al-55) at the ends of, or at breaks in, the chains. These topologies also explain the lower abundances of μ_1_-OH (Al-6) and μ_1_-OH (Al-5) relative to μ_1_-OH (Al-4). Upon hydroxylation of one such Al^V^ or Al^IV^ environment, μ_1_-OH (Al-6) or μ_1_-OH (Al-5) are formed, respectively, with two neighbouring coordinatively unsaturated aluminium sites. A small displacement of the oxygens of these hydroxyls generates a μ_2_ aluminol, which is energetically more favourable. Chains of μ_1_-OH therefore give rise to corresponding chains of μ_1_-OH (Al-6) or μ_1_-OH (Al-5), which also do not correspond to the ^1^H signals at lower *δ*. Trace A shows autocorrelations between μ_1_-OH, while trace G demonstrates correlations between H that are hydrogen-bond donors and μ_1_-OH. The intensity of these cross peaks decreases significantly with recoupling time (see Fig. S10.4 of the ESI†), suggesting close proximity of the hydrogen-bond donors H and μ_1_-OH on the surface. Trace H confirms the autocorrelation of two hydrogen-bond donors (a signal which also decreases with recoupling time), suggesting this most likely arises from water. The only autocorrelation that is not observed is for μ_3_-OH, most likely because of their low overall concentration.

## Conclusions

In this work we have used a multinuclear NMR crystallographic approach, combining periodic planewave DFT calculations with experimental NMR spectroscopy of isotopically enriched materials, to probe the detailed atomic-level structure of γ-Al_2_O_3_. Although each of these approaches has been used in previous work, the combination of all three in a single study here has demonstrated the complexity of the bulk material and range of surface species that may be present, and the challenge of unambiguously assigning (and quantifying) the experimental signals observed (an approach on which some previous literature conclusions have depended). This complexity has potentially limited prior analysis, and the insight obtained in this work provides new opportunities to interrogate this important system and related materials using NMR crystallography.

Comparison of experiment and computation suggested that the bulk structure of γ-Al_2_O_3_ contained Al cations primarily in “spinel-like” sites, with roughly equal numbers of alternating Al^VI^ and Al^IV^ vacancies in disordered “chains” throughout. This contrasts with some previous work where Al vacancies were thought to occur exclusively on Al^VI^ sites, with a maximum separation between them. The use of a larger unit cell for the calculations enabled a wide range of local environments to be generated, and the inclusion of Al^IV^ vacancies produces many new types of ^17^O signals, and notably O^III^ sites with very similar parameters to O^IV^ signals. This highlights potential problems with previous work, where many conclusions relied on an unambiguous assignment (and quantification) of O^III^ and O^IV^ signals and correlations of these with other species to determine local structure. There was no evidence for the presence of H in the bulk structure of γ-Al_2_O_3_, or for any significant levels of O vacancies.

While catalysis is often a surface phenomenon, the surface structure is inherently related to the position, type and ordering of Al vacancies found in the bulk. Applying an NMR crystallographic approach to three separate surfaces made from a set of our structural models with different cation vacancies and different levels of hydration, enabled the prediction of ^17^O NMR spectra for surface, sub surface and bulk species. This showed that even at the third oxide layer from the surface many different types of O species are formed owing to the different coordination numbers of the neighbouring Al cations, many of which are now reduced by the cleavage, providing additional support for the assignment and interpretation of the complex resonances in the ^17^O NMR spectra.

Relaxation measurements highlighted the involvement of protonated O species in dynamic surface processes and their interactions with water, with the rapid relaxation seen accounting for the absence of these signals in STMAS (and MQMAS) spectra. Through VT, CP and TRAPDOR experiments, and by comparison to DFT calculations, it was possible to assign the two signals seen, with the sharper and broader resonances attributed to more accessible surface sites that often interact strongly with water and less accessible aluminols, respectively. ^17^O-^27^Al D-HMQC spectra showed that all O signals correlate to Al^IV^ and Al^VI^ species but, somewhat surprisingly, J-HMQC experiments suggested that the correlations are much stronger with Al^IV^ than with Al^VI^. However, this is likely to result from the different *J* couplings, with O–Al^VI^ couplings much smaller (3–10 Hz) than O–Al^IV^ couplings, reflecting the different bond lengths, rather than a difference in the presence of these atom pairs.

DFT-predicted ^1^H NMR parameters confirmed the ^1^H shift generally increases with denticity (in agreement with previous work), and that, although a wide range of shifts are seen depending on the coordinated atoms, there is little direct dependence of the ^1^H shift on the Al coordination number. Spectral assignments were also supported by ^1^H–^27^Al RESPDOR experiments, which identified spectral components resulting from μ_1_, μ_2_ and μ_3_ aluminols. Combining these with ^1^H–^27^Al D-HMQC experiments showed that (i) μ_1_ aluminols are more likely to be bound to Al^IV^, (ii) μ_2_ aluminols are coordinated to all three types of Al, but with a higher proportion bound to similar types of Al, *e.g.*, μ_2_-OH (Al-44), μ_2_-OH (Al-56) and μ_2_-OH (Al-66) and (iii) μ_3_ aluminols are most likely bound to higher coordinated Al species, *e.g.*, μ_3_-OH (Al-666) and μ_3_-OH (Al-665). DQ MAS spectroscopy confirmed no types of aluminols exist exclusively in isolation but showed that the closest proximities are between bridging aluminols coordinated to Al^IV^ and/or Al^V^ species, reflecting the likely topology of the surfaces.

The combination of multinuclear NMR spectroscopy with both isotopic enrichment and DFT calculations has provided more detailed insight into both the bulk and surface structure of γ-Al_2_O_3_. The complexity of both bulk and surface structures that this reveals highlights the challenges faced by any study, and the need to combine approaches to obtain a much fuller picture. The different results observed in this work from some previous studies may reflect the natural advances in both computation and experiment over the years but are also likely to result from the varied nature of aluminas depending on the route by which they are synthesised and how they have been treated or stored. While this adds to the challenge of control for catalytic applications, it also offers an exceptional opportunity to fine tune the properties of the surfaces and their modification if the detailed atomic-level structure of these materials can be understood. The insight achieved here will provide a basis for future work both on the reactivity of aluminas themselves and on their modified surfaces.

## Data availability

Detailed experimental parameters, structural models used for DFT calculations, further ^17^O, ^27^Al and ^1^H NMR spectra and calculated ^27^Al–^17^O *J* couplings are given in the ESI.† The research data (and/or materials) supporting this publication can be accessed at https://doi.org/10.17630/5020123c-d803-4bfd-8228-3e2507f077e4.^87^ In order to meet institutional and research funder open access requirements, any accepted manuscript arising shall be open access under a Creative Commons Attribution (CC BY) reuse licence with zero embargo.

## Author contributions

SEA and PBW co-conceived and funded the project and had oversight and supervision of the work. MLB carried out the experimental and computational work. DMD supported the acquisition, analysis and curation of the NMR data and contributed to the preparation of the figures. SEA wrote the initial manuscript and all authors contributed to the final draft.

## Conflicts of interest

There are no conflicts of interest to declare.

## Supplementary Material

SC-016-D5SC01198A-s001
