# Supplementary material for: Insight into the atomic-level structure of γ-alumina using a multinuclear NMR crystallographic approach
Source: Chem Sci. 2025 Mar 12;16(18):7695–710. doi: 10.1039/d5sc01198a (PMC11962746; doi:10.1039/d5sc01198a)
Supplement: SC-016-D5SC01198A-s001 [file SC-016-D5SC01198A-s001.pdf]

Supplementary Information (SI) for Chemical Science.
This journal is © The Royal Society of Chemistry 2025

Insight into the Atomic-Level Structure of γ-Alumina Using a Multinuclear NMR Crystallographic Approach

M. Bonifac Legrady,[1,2] Daniel M. Dawson,[1] Paul B. Webb[1] and Sharon E. Ashbrook,[1*]

[1] School of Chemistry, EaStCHEM and Centre of Magnetic Resonance, University of St Andrews, North Haugh, St Andrews KY16 9ST, UK

[2] Current address: School of Chemistry, University of Southampton, University Road, Southampton, SO17 1BJ, UK

Supporting Information

S1. Detailed experimental parameters

S2. Characterisation of γ-Al_2O_3 after post-synthetic exchange with $^{17}O_2$(g)

S3. Structural models used for DFT calculations of bulk γ-Al_2O_3

S4. Structural models of bulk γ-Al_2O_3 containing ^{1}H

S5. Structural models of bulk γ-Al_2O_3 containing oxygen vacancies

S6. Structural models of surfaces of γ-Al_2O_3

S7. ^{17}O MAS and CP MAS NMR of γ-Al_2O_3

S8. ^{27}Al MAS and MQMAS NMR of γ-Al_2O_3

S9. ^{27}Al-^{17}O calculated J couplings

S10. ^{1}H MAS NMR of γ-Al_2O_3

S11. References

S1. Detailed experimental parameters

Figure 2a

Nucleus: ^{17}O

Sample: γ-$Al_2{}^{17}O_3$(500 °C), enriched by post-synthetic exchange with $^{17}O_2$ (g) at 500 °C for 48 h

Pulse sequence: single pulse MAS

Magnetic fields: 9.4, 14.1 and 20.0 T

MAS rates: 14, 14 and 20 kHz

Other parameters: 4096 transients averaged with a recycle interval of 0.5 s (9.4 T), 10240 transients averaged with a recycle interval of 0.5 s (14.1 T) and 4096 transients averaged with a recycle interval of 0.5 s (20.0 T)

Figure 2b

Nucleus: ^{17}O

Sample: γ-$Al_2{}^{17}O_3$(950 °C), enriched by post-synthetic exchange with $^{17}O_2$ (g) at 950 °C for 14 h (after prior heating at 800 °C for 6 h)

Pulse sequence: split-t_1 shifted-echo double-quantum filtered STMAS pulse sequence, with rf nutation rates of 100 kHz and 12.5 kHz for hard and soft pulses, respectively

Magnetic field: 20.0 T

MAS rate: 30 kHz

Other parameters: 1664 transients averaged for each of 100 t_1 increments of 43.05 μs, using a 1 s recycle interval (total experimental time 1.9 days)

Figure 7a

Nucleus: ^{17}O

Sample: γ-$Al_2{}^{17}O_3$(500 °C), enriched by post-synthetic exchange with $^{17}O_2$ (g) at 500 °C for 72 h

Pulse sequence: single pulse and cross polarisation from ^1H with τ_{CP} of 400 μs

Magnetic field: 20.0 T

MAS rate: 20 kHz

Other parameters: for both spectra 4096 transients averaged using a 0.5 s recycle interval

Spectra are normalised such that the intensity of the broad component is the same

Figure 7b

Nucleus: ^{17}O

Sample: γ-$Al_2{}^{17}O_3$(500 °C), enriched by post-synthetic exchange with $^{17}O_2$ (g) at 500 °C for 72 h

Pulse sequence: single pulse at temperatures shown

Magnetic field: 20.0 T

MAS rate: 20 kHz

Other parameters: 3664 (–60 °C), 2048 (RT) and 2800 (100 °C) transients averaged using a 0.5 s recycle interval

Spectra are scaled such that all have the same intensity at 60 ppm to facilitate comparison of the lineshapes

Figure 7c

Nucleus: ^{17}O

Sample: γ-$Al_2{}^{17}O_3$(500 °C), enriched by post-synthetic exchange with $^{17}O_2$ (g) at 500 °C for 72 h

Pulse sequence: TRAPDOR experiment with (green) and without (blue) recoupling pulses, shown along with the corresponding difference spectrum (red)

Magnetic field: 14.1 T

MAS rate: 14 kHz

Other parameters: 256 transients averaged using a 0.5 s recycle interval with TRAPDOR recoupling (80 kHz) for τ_{rec} = 16 τ_R. The HS pulse duration was 1.1 ms.

Figure 8a

Nucleus: ^{27}Al

Sample: γ-Al_2O_3, hydrated and dehydrated at 300 and 700 °C

Pulse sequence: single pulse MAS

Magnetic field: 9.4 T

MAS rate: 14 kHz

Other parameters: 64, 64 and 128 transients were averaged using a 3 s, 3 s and 1 s recycle interval for spectra of hydrated γ-Al_2O_3, and γ-Al_2O_3 dehydrated (300 °C) and dehydrated (700 °C), respectively

Spectra are shown normalised to the intensity of the Al(VI) signal

Figure 8b

Nucleus: ^{27}Al

Sample: γ-Al_2O_3, hydrated and dehydrated at 300 and 700 °C

Pulse sequence: Cross polarisation from ^{1}H with τ_{CP} of 800 μs

Magnetic field: 9.4 T

MAS rate: 14 kHz

Other parameters: 3584, 7168 and 8192 transients were averaged using a 1 s recycle interval for spectra of hydrated γ-Al_2O_3, and γ-Al_2O_3 dehydrated (300 °C) and dehydrated (700 °C), respectively

Spectra are shown normalised to the intensity of the Al(VI) signal

Figure 8c

Nucleus: ^{27}Al

Sample: γ-Al_2O_3, hydrated and dehydrated at 300 °C

Pulse sequence: z-filtered MQMAS pulse sequence, with rf nutation rates of ~90-100 kHz and ~7-9 kHz for hard and soft pulses

Magnetic field: 14.1 T

MAS rate: 14 kHz

Other parameters: 48 transients averaged for each of 104 t_1 increments of 25.0 μs, using a 1 s recycle interval (total experimental time 1.4 h) for hydrated material, and 144 transients averaged for each of 120 t_1 increments of 17.86 μs, using a 1 s recycle interval (total experimental time 4.8 h) for dehydrated material

Figure 8d

Nucleus: $^{27}Al-^{17}O$

Sample: $\gamma-Al_2{}^{17}O_3$(950 °C), enriched by post-synthetic exchange with $^{17}O_2$ (g) at 950 °C for 14 h (after prior heating at 800 °C for 6 h)

Pulse sequence: HMQC without (J-HMQC) and with (D-HMQC) 0.9 ms of $SR4^2_1$ recoupling

Magnetic field: 20.0 T

MAS rate: 20 kHz

Other parameters: 1664 transients averaged for each of 40 (D-HMQC) and 60 t_1 (J-HMQC) increments of 50 μs, using a 1 s recycle interval, for evolution times of 0.9 ms (D-HMQC) and 4.5 ms (J-HMQC)

Figure 9a

Nucleus: ^{1}H

Sample: : $\gamma-Al_2O_3$ hydrated, and dehydrated at 300 °C and 550 °C

Pulse sequence: DEPTH

Magnetic field: 14.1 T

MAS rate: 14 kHz

Other parameters: 800, 128 and 16 transients were averaged using a 1 s recycle interval, for hydrated and dehydrated (300 °C) and dehydrated (550 °C) materials, respectively

Figure 9b

Nucleus: ^{1}H

Sample: $\gamma-Al_2O_3$ dehydrated at 550 °C

Pulse sequence: DEPTH

Magnetic field: 14.1 T

MAS rate: 20 kHz

Other parameters: 256 transients were averaged using a 3 s recycle interval

Figure 10a

Nucleus: $^{1}H/^{27}Al$

Sample: γ-Al_2O_3 dehydrated at 550 °C

Pulse sequence: DEPTH

Magnetic field: 14.1 T

MAS rate: 40 kHz

Other parameters: 64 transients were averaged at a temperature of 266 K using a 10 s recycle interval

Figure 10b

Nucleus: $^{1}H/^{27}Al$

Sample: γ-Al_2O_3 dehydrated at 550 °C

Pulse sequence: RESPDOR (using R1 2_3^5 recoupling)

Magnetic field: 14.1 T

MAS rate: 40 kHz

Other parameters: 64 transients were averaged at a temperature of 266 K using a 3 s recycle interval for each of 26 recoupling times separated by 25 μs

Figure 11a

Nucleus: $^{1}H/^{27}Al$

Sample: γ-Al_2O_3 dehydrated at 550 °C

Pulse sequence: D-HMQC (using TRAPDOR recoupling)

Magnetic field: 14.1 T

MAS rate: 40 kHz

Other parameters: 200 transients averaged for each of 120 t_1 increments of 25 μs, using a 3 s recycle interval and a TRAPDOR recoupling time of 375 μs

Figure 11b

Nucleus: ^{1}H

Sample: γ-Al_2O_3 dehydrated at 550 °C

Pulse sequence: DQ MAS (using BABA recoupling)

Magnetic field: 14.1 T

MAS rate: 40 kHz

Other parameters: 80 transients averaged for each of 200 t_1 increments of 25 μs, using a 1 s recycle interval and a recoupling time of 25 μs

S2. Characterisation of γ-Al_2O_3 after post-synthetic exchange with $^{17}O_2$(g)

A sample of γ-Al_2O_3 (γ-Al_2O_3(950 °C)) was enriched post synthesis by heating with 70% $^{17}O_2$(g) for at 800 °C for 6 h and, subsequently, at 950 °C for 14 h. From powder XRD this sample contains small impurities of δ-Al_2O_3 and θ-Al_2O_3, although the predominant phase remains γ-Al_2O_3. The ^{17}O MAS and 3QMAS NMR spectra of this sample are compared with those of a sample of γ-Al_2O_3 enriched at 500 °C γ-Al_2O_3(500 °C) for 72 h in Figure S2.1.

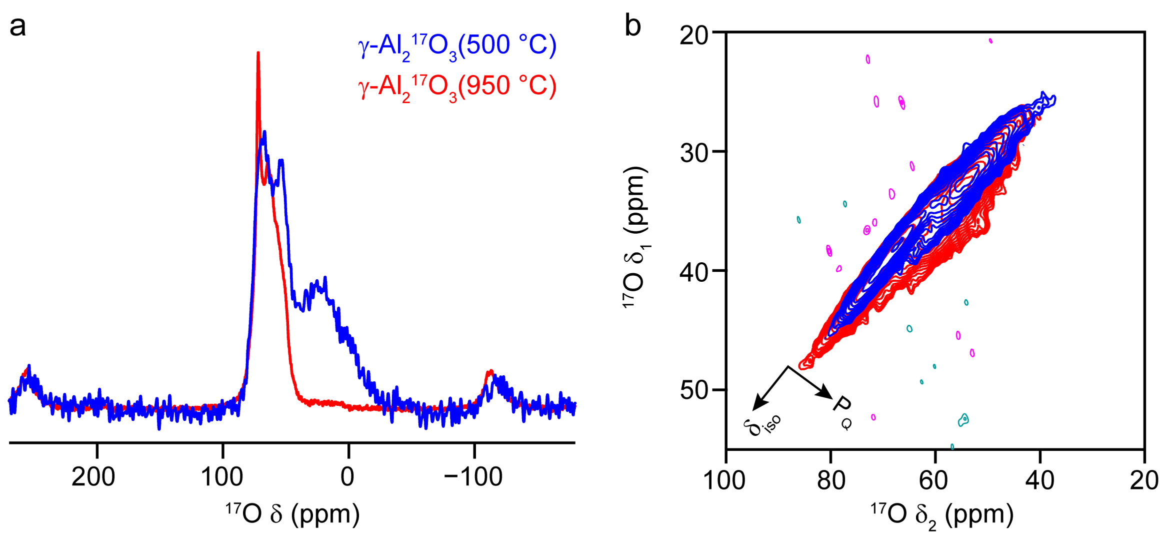

Figure S2.1. ^{17}O (18.8 T, 20 kHz) (a) MAS and (b) 3QMAS spectra of γ-$Al_2{}^{17}O_3$(500 °C) (blue) and γ-$Al_2{}^{17}O_3$(950 °C) (red). In (a), spectra were acquired by averaging 4096 transients using a 1 s recycle interval. In (b), spectra were acquired using a z-filtered triple-quantum MAS pulse sequence and are the result of averaging 7704 (blue) or 9678 (red) transients for each of 80 t_1 increments of 50 μs, with a recycle interval of 0.5 s.

As seen in Figure S2.1a, the use of a higher enrichment temperature leads to a significantly higher level of enrichment for non-protonated O sites, although the differences seen in the relative intensities of the two signals suggests that enrichment is seen deeper in the bulk structure as the temperature increases. The intensity of the signals from the protonated O species is lower.

The 3QMAS NMR spectra in Figure S2.1b show that the ^{17}O signals appear at similar positions in the two samples (note that the signals from the protonated O species are not seen in MQMAS and STMAS spectra owing to very rapid relaxation – see the main text for a more detailed discussion). The ridge centred at higher δ_1 values extends towards higher δ (i.e., higher δ_{iso}) for γ-$Al_2{}^{17}O_3$(950 °C), most likely as a result of the deeper penetration of the enrichment into the bulk at higher temperatures. The second ridge (centred at lower δ_1 values) extends to lower δ_1 shifts for γ-$Al_2{}^{17}O_3$(950 °C). In Figure S2.2, the centre-of-gravity of ^{17}O signals of δ-Al_2O_3 and θ-Al_2O_3 (predicted using DFT calculations of structural models from the literature) are overlaid with the experimental DQF-STMAS spectra of γ-$Al_2{}^{17}O_3$(950 °C). Recent work on δ-Al_2O_3 has shown that it is perhaps best described as a structural intergrowth of two main crystallographic variants, δ_1-Al_2O_3 and δ_2-Al_2O_3.[S1] The structure of θ-Al_2O_3 is generally accepted to be isomorphic to β-Ga_2O_3.[S2] Figure S2.2 shows that the calculated O^{III}(664) sites in δ_1-Al_2O_3 and δ_2-Al_2O_3 do not match the centre-of-gravity of the lower ridge in the experimental spectrum but are shifted towards higher P_Q (higher δ_1) values, suggesting this is perhaps the cause of the slight broadening of this ridge in the experimental STMAS spectrum. The calculated δ_1 and δ_2 values for O^{III}(644) match reasonably well with the experimentally observed shoulder on the lower ridge, which is also not observed in the high-resolution spectra of γ-$Al_2{}^{17}O_3$(500 °C) at any field.

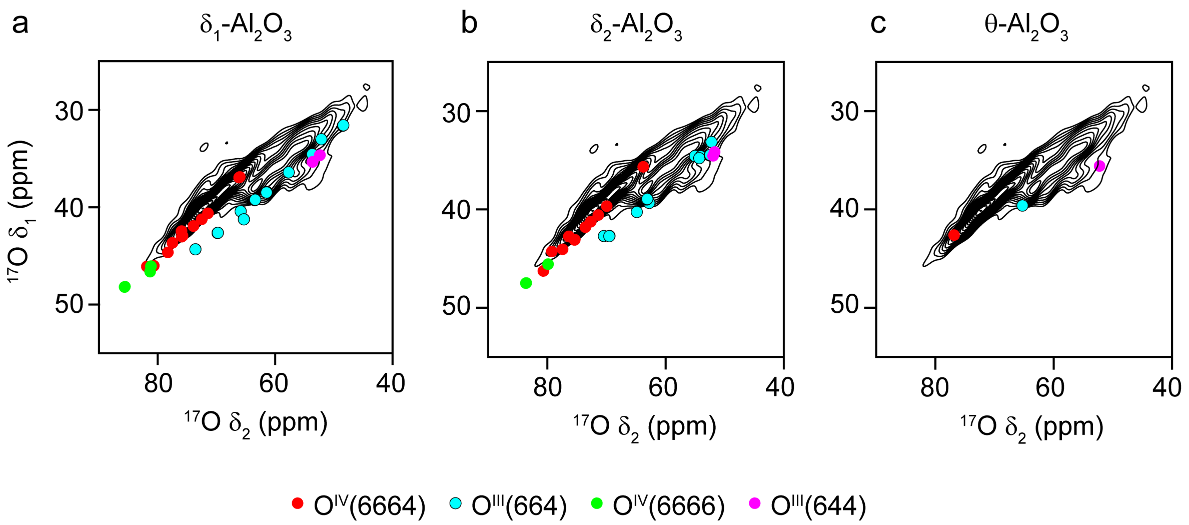

Figure S2.2. Plots showing the predicted (δ_1, δ_2) shifts corresponding to the centre-of-gravity of the signals of different O environments in (a) δ_1-Al_2O_3, (b) δ_2-Al_2O_3 and (c) θ-Al_2O_3, as predicted by DFT calculations, overlaid onto the ^{17}O (20.0 T, 30 kHz) DQF-STMAS spectrum of γ-$Al_2{}^{17}O_3$(950 °C) shown in Figure 2b of the main text.

S3. Structural models used for DFT calculations of bulk γ-Al_2O_3

Spinel and non-spinel models (Paglia)

Figure S3.1 shows the lowest energy structural models of γ-Al_2O_3 for each type of unit cell considered by Paglia[S3] (3 × 1 × 1 supercells of the cubic ($Fd\bar{3}m$) and tetragonal ($I41/amd$) unit cells), with the different types of O environments indicated by differing colours. These can be separated into "spinel" sites (i.e., local environments that would occur in structures where all of the cations are restricted to spinel positions) and "non-spinel" sites (i.e., local environments that would not occur if this restriction was in place).

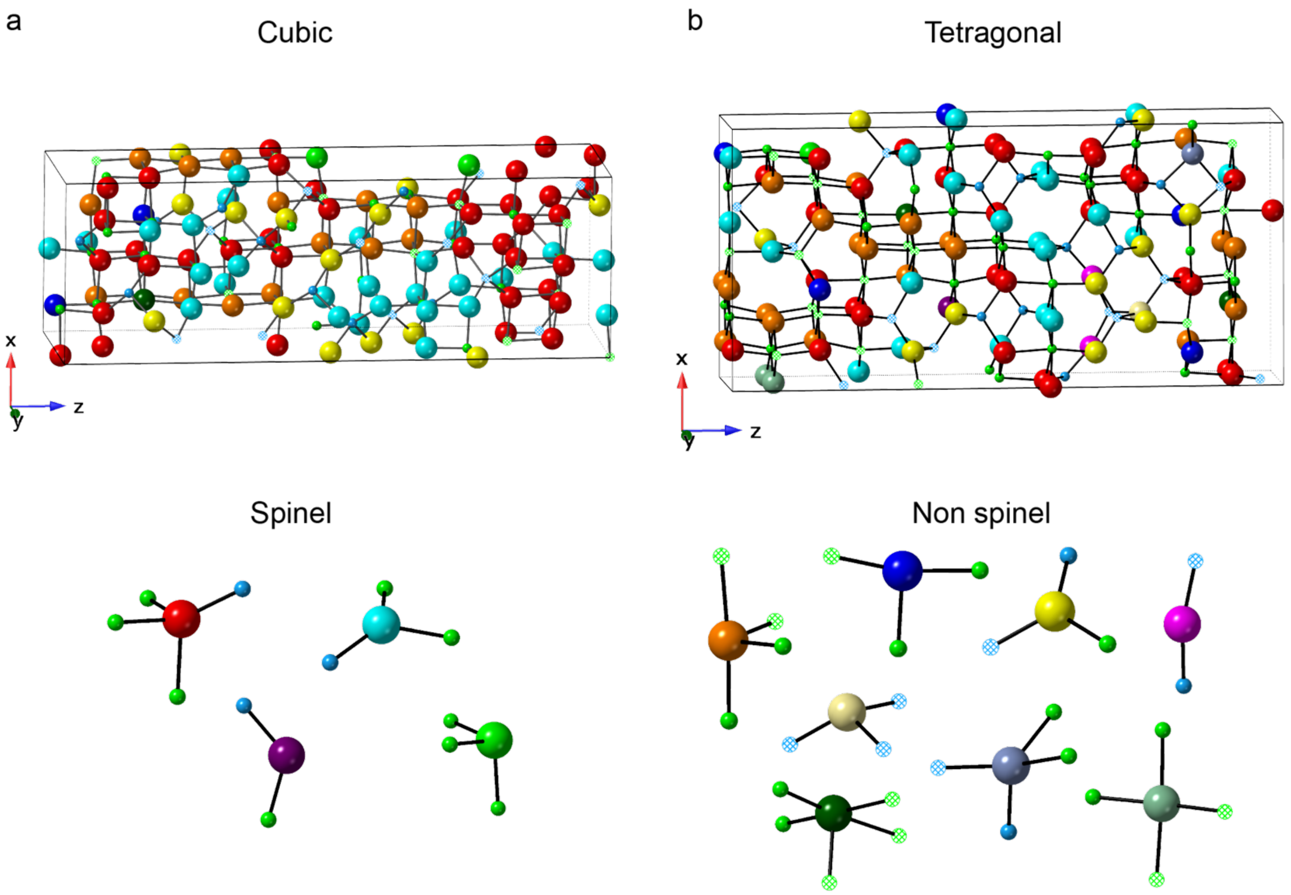

Figure S3.1. The two non-spinel models (3 × 1 × 1 supercells of (a) cubic and (b) tetragonal unit cells) of bulk γ-Al_2O_3 proposed by Paglia,[S3] used for DFT prediction of NMR parameters, with differing O local environments highlighted by colour. See Figure 3a of the main text for the corresponding DFT-predicted parameters.

Bulk spinel models used for DFT calculations

Figures 3.2-3.5 show the structures of the spinel-based models used for DFT calculations. A complete set of unit cell parameters and atomic positions can be found in https://doi.org/10.17630/5020123c-d803-4bfd-8228-3e2507f077e4.

Model SP1: Ordered Al^{VI} vacancies with maximum separation between them

a

b

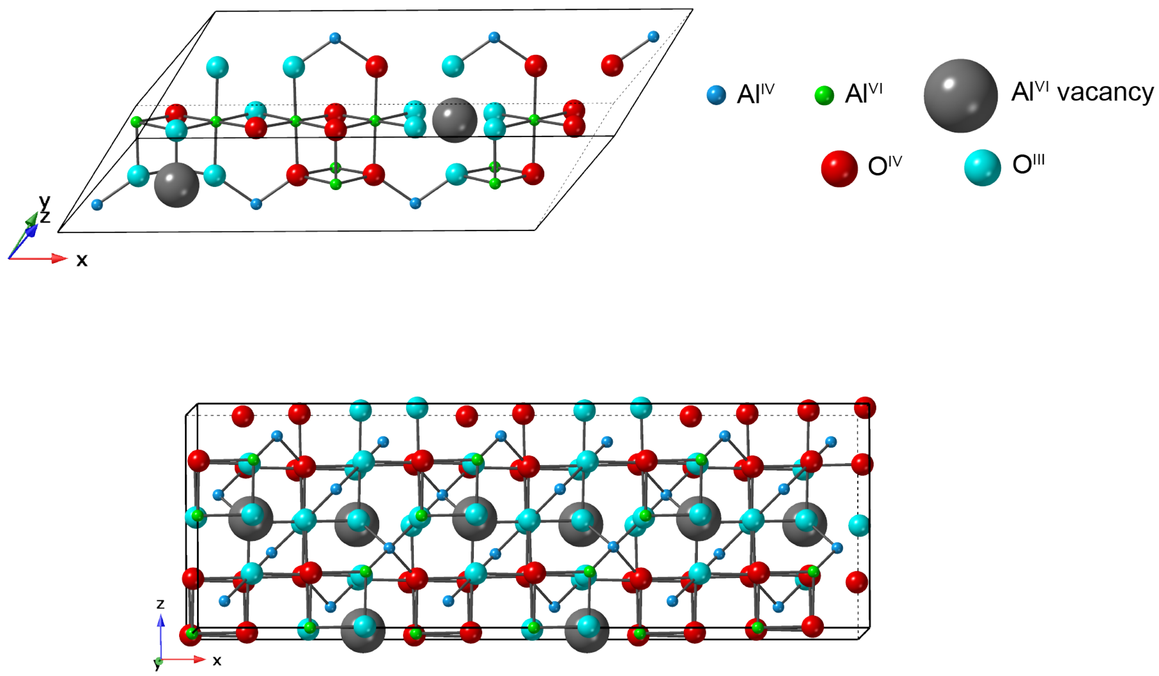

Figure S3.2. Schematics of the structure (and position of the cation vacancies) in (a) the 3 × 1 × 1 supercell of the primitive *Fd*$\bar{3}$*m* unit cell and (b) the 3 × 1 × 1 supercell of the *Fd*$\bar{3}$*m* cubic spinel unit cell (Model SP1) of bulk γ-Al_2O_3, both generated assuming only Al^{VI} vacancies are present, and that there is maximum separation between these, with differing O local environments highlighted by colour. See Figure 4 of the main text for the corresponding DFT-predicted parameters for Model SP1.

Model SP2: Al^{VI} vacancies with varying separation

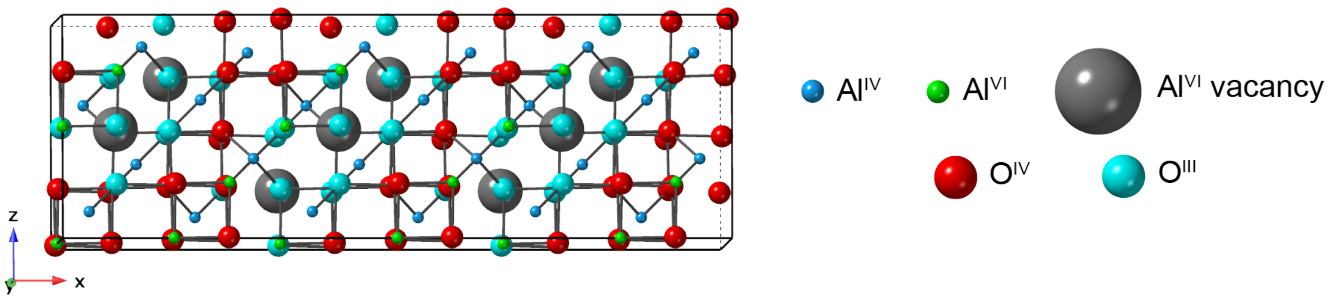

Figure S3.3. A spinel-based model (Model SP2) of bulk γ-Al_2O_3 generated from a 3 × 1 × 1 supercell of the cubic $Fd\bar{3}m$ spinel unit cell assuming only Al^{VI} vacancies are present (now with varying separation), with differing O local environments highlighted by colour. See Figure 4 of the main text for the corresponding DFT-predicted parameters.

Models SP3-5: Al^{VI} and Al^{IV} vacancies with varying separation

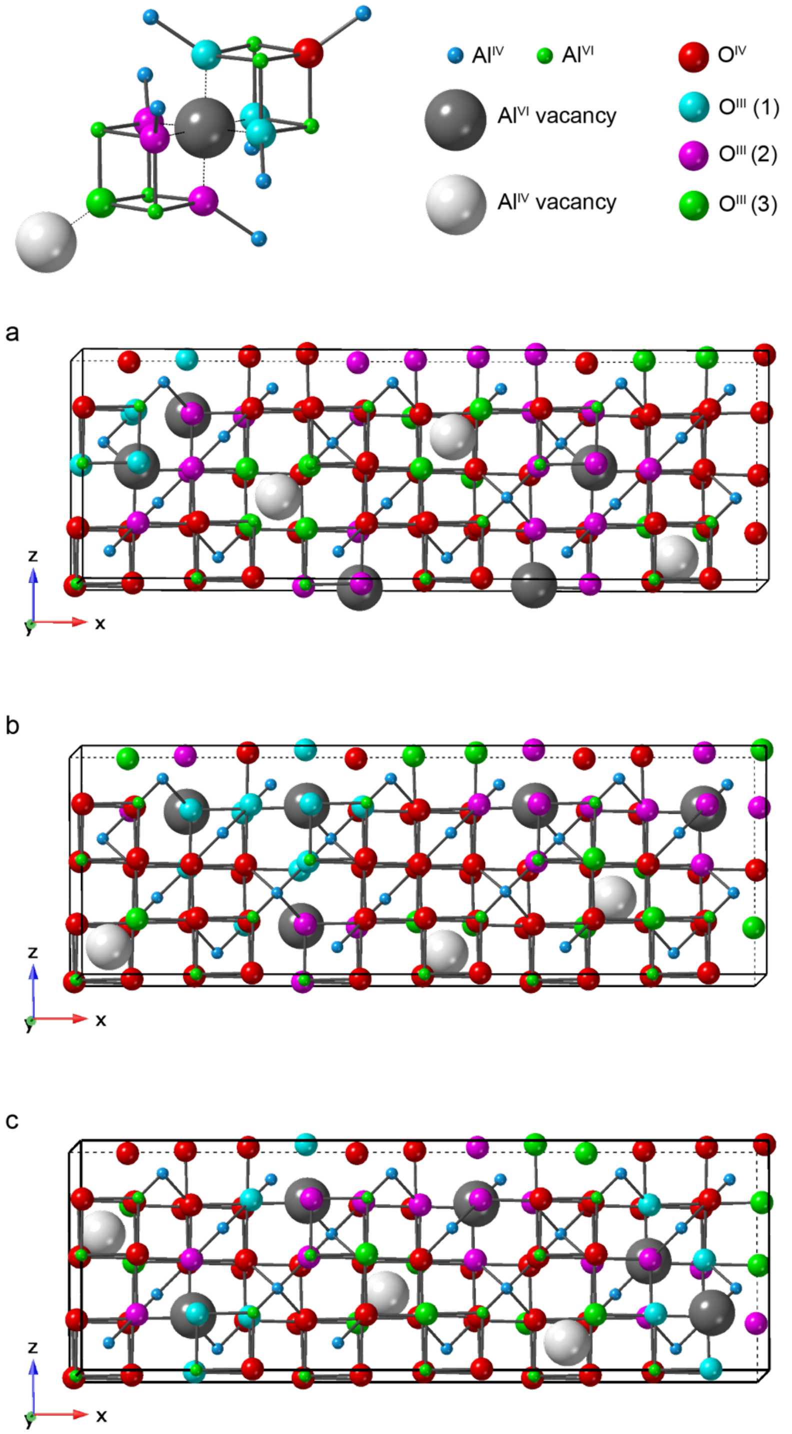

Figure S3.4. Three different spinel-based models (Models SP3-5 for (a), (b) and (c), respectively) of bulk γ-Al_2O_3 generated from a 3 × 1 × 1 supercell of the $Fd\bar{3}m$ cubic spinel unit cell with both Al^{VI} and Al^{IV} vacancies, and with varying separation between these, with differing O local environments highlighted by colour (see key). See Figure 5 of the main text for the corresponding DFT-predicted parameters.

Models SP6-8: Structures with different arrangements of alternating Al^{VI} and Al^{IV} vacancies

Figure S3.5 shows the overlay of DFT-predicted (δ_1, δ_2) centre-of-gravity shifts for O environments in three different spinel-based models (Models SP6-8) of bulk γ-Al_2O_3 generated assuming alternating Al^{VI} and Al^{IV} vacancies, with the ^{17}O (20.0 T, 30 kHz) DQF-STMAS spectrum of γ-$Al_2{}^{17}O_3$(950 °C) from Figure 2b of the main text. Structural models with alternating Al^{VI} and Al^{IV} vacancies must, by definition, contain a line of Al^{IV} - Al^{VI} - Al^{IV} vacancies (see Figure 4a of the main text), although the next Al^{VI} vacancy can then be in the direction of any of the three neighbouring O atoms. In a real crystal this would generate complex vacancy “trajectories” and lead to many different O species in the resulting disordered solid. However, when limited to a 160 atom cell this results in ordered systems, leading to a relatively small number of distinct O species, which do not represent well the range that would appear in reality. Model SP6 was generated from the spinel-based 3 × 1 × 1 supercell used so far, and results in a very ordered structure (where the vacancies appear in parallel zig zag lines – see Figure S3.6). Model SP8 was generated by transforming the original cubic spinel cell such that the yz face corresponds to the (110) plane of the original cubic cell, and a 3 × 1 × 1 supercell was then constructed from this, prior to the introduction of the vacancies. This leads to a structure exhibiting antiparallel zig-zag lines (see Figure S3.6). Model SP7 was generated from a 3 × 2 × 2 supercell of the primitive cell prior to introducing vacancies. The yz face of this structure is in the (111) plane of the cubic cell, generating a structure that contains a mixture of parallel and antiparallel zig zag lines of vacancies (see Figure S3.6). In these models, the O^{III}(664) sites shown in magenta are either very close to or very far away from a third Al vacancy (and have very low or very high δ_{iso} values, respectively). In a real material, a range of distances would be expected as a result of the more disordered longer-range structure, giving rise to intermediate δ_{iso} values that should agree better with experiment.

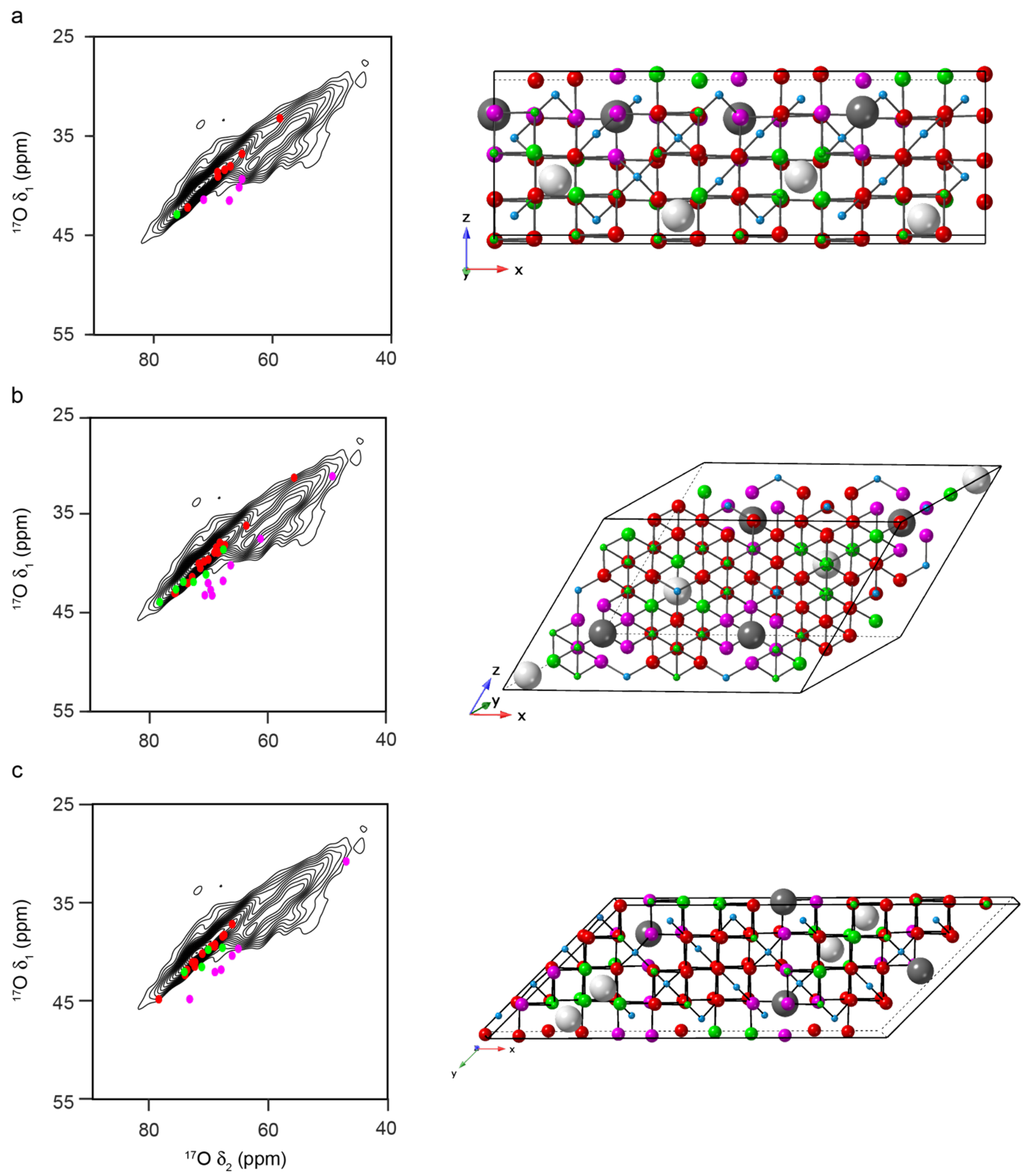

Figure S3.5. Plots showing the overlay of (δ_1, δ_2) centre-of-gravity shifts predicted (using DFT) for different O environments of three different spinel-based models (Models SP6-8) of bulk γ-Al_2O_3 generated from supercells of the (a, c) cubic and (b) primitive spinel unit cell using different arrangements of alternating Al^{VI} and Al^{IV} vacancies, with the ^{17}O (20.0 T, 30 kHz) DQF-STMAS spectrum of γ-$Al_2{}^{17}O_3$(950 °C) from Figure 2b of the main text. See Figure S3.6 for schematics of the vacancy distribution found in each structure. For key to colours see Figure S3.4.

a

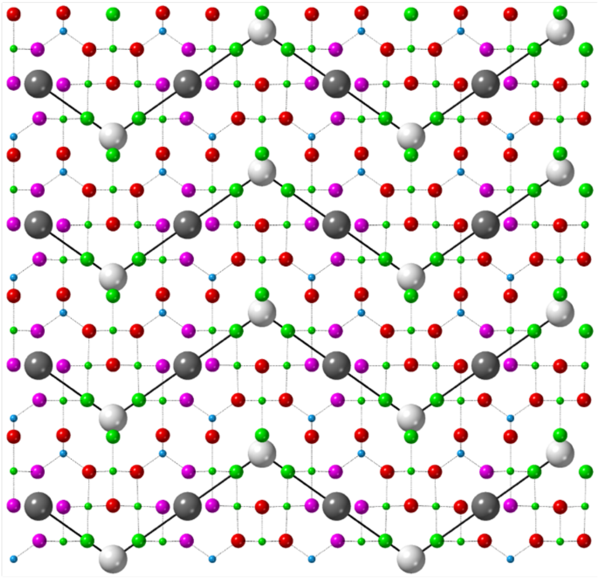

b

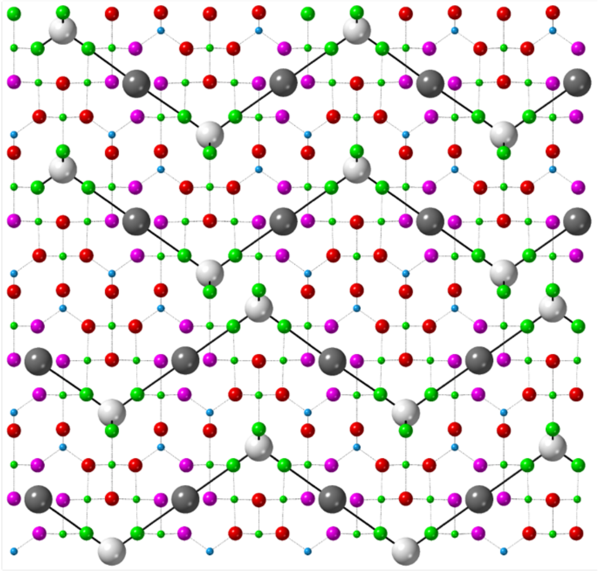

c

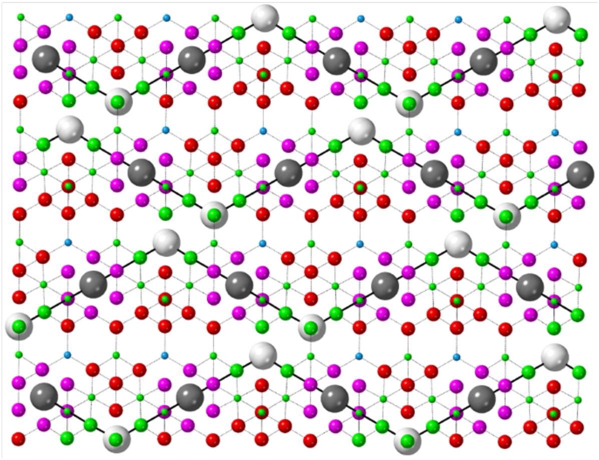

Figure S3.6. Distribution of the vacancies in the three different spinel-based models (Models SP6-8) of bulk γ-Al_2O_3 generated using alternating Al^{VI} and Al^{IV} vacancies, shown in Figure S3.5. For key to colours see Figure S3.4.

S4. Structural models of bulk γ-Al_2O_3 containing ^1H

Models SPH1-4 were generated from Model SP6 (see above) by replacing one Al^{3+} cation with three ^1H. For Models SPH1 and SPH2 one of the chemically inequivalent Al^{VI} species is replaced, whereas for SPH3 and SPH4 the replacement is of one of the inequivalent Al^{IV} species. Figure S4.1 shows the overlay of DFT-predicted (δ_1, δ_2) centre-of-gravity shifts for O environments in these four models of bulk γ-Al_2O_3 with the ^{17}O (20.0 T, 30 kHz) DQF-STMAS spectrum of γ-$Al_2{}^{17}O_3$(950 °C) from Figure 2b of the main text. Little evidence of signals from the protonated O species (predicted using DFT to be at much lower δ) is seen experimentally. (Note that although no experimental STMAS signals are seen for the protonated surface O species owing to the rapid relaxation, dynamics are expected to be substantially reduced for bulk H species).

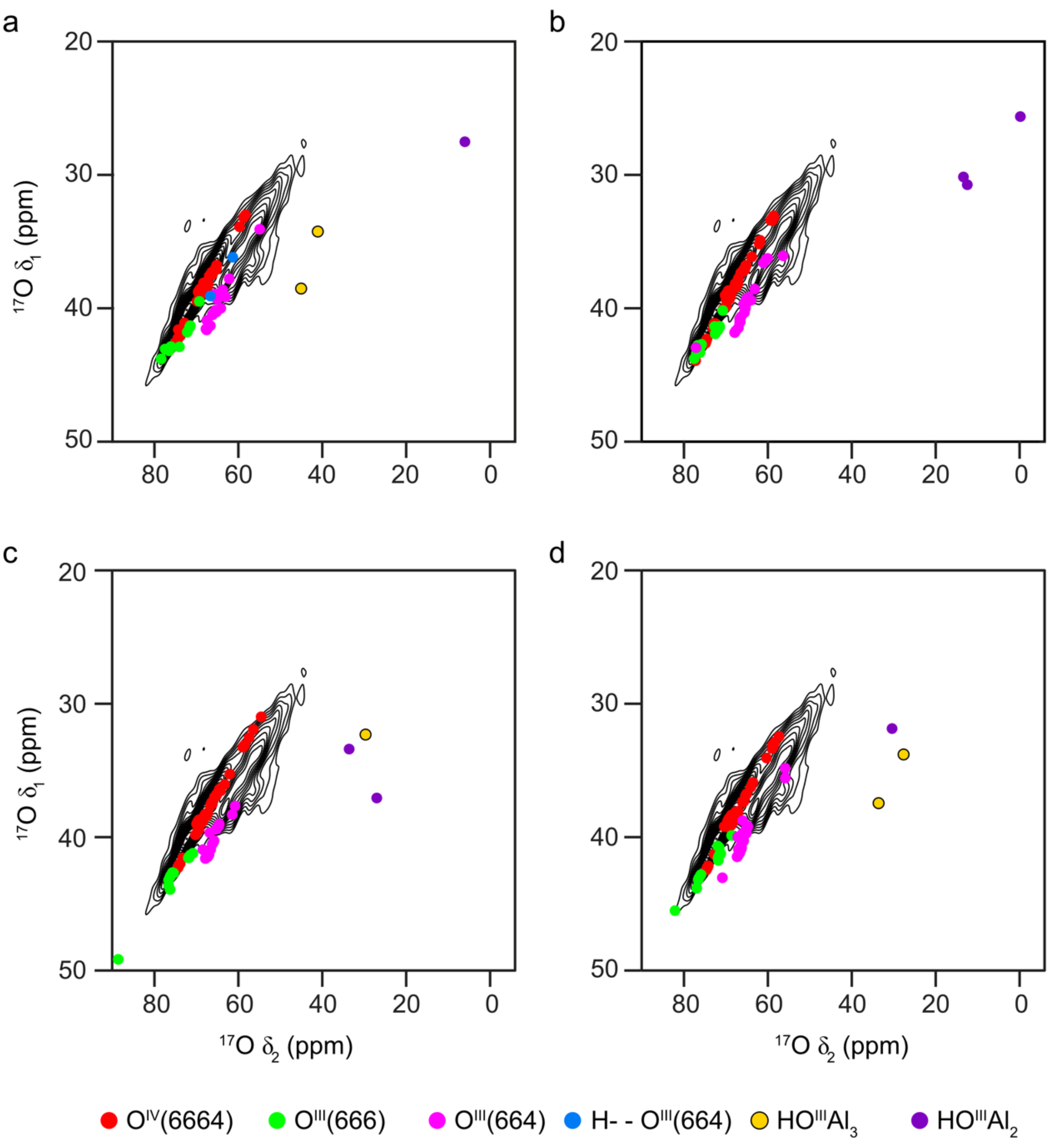

Figure S4.1. Plots showing the overlay of (δ_1, δ_2) centre-of-gravity shifts predicted (using DFT) for different O environments of three different models (Models SPH1-4, in (a), (b), (c) and (d), respectively) of bulk γ-Al_2O_3 generated from Model SP6 by replacing one Al^{3+} cation with three ^1H, with the ^{17}O (20.0 T, 30 kHz) DQF-STMAS spectrum of γ-$Al_2{}^{17}O_3$(950 °C) from Figure 2b of the main text.

Figure S4.2 shows the DFT-predicted ^1H δ_{iso} values for Models SPH1-4 of bulk γ-Al_2O_3, overlaid with a ^1H (14.1 T, 20 kHz) DEPTH MAS NMR spectrum of dehydrated γ-$Al_2{}^{17}O_3$(550 °C), again suggesting little evidence that γ-Al_2O_3 contains a significant amount of ^1H in the bulk structure.

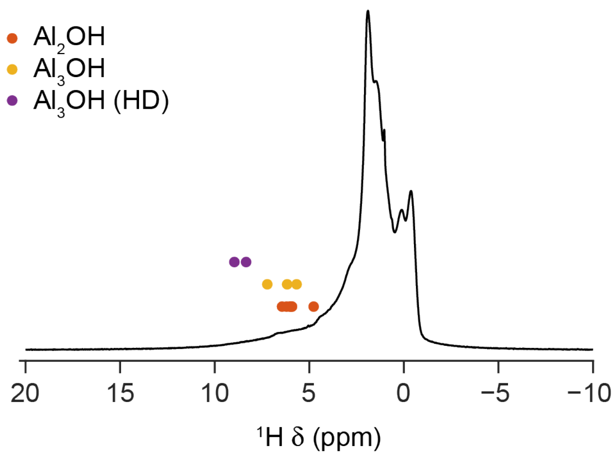

Figure S4.2. Overlay of DFT-predicted ^{1}H δ_{iso} values from Models SPH1-4 of bulk γ-Al_2O_3 generated from Model SP6 by replacing one Al^{3+} cation with three H^+, with a ^{1}H (14.1 T, 20 kHz) DEPTH spectrum of dehydrated γ-$Al_2{}^{17}O_3$(550 °C). The spectrum is the result of averaging 256 transients using a 3 s recycle interval.

S5. Structural models of bulk γ-Al_2O_3 containing oxygen vacancies

Models SPV1-2 were generated from Model SP6 the removal of 3 O^{2-} anions and 2 Al^{3+} cations. In Model SPV1 the O vacancies are more remote in the structure, whereas in Model SPV2 an "Al_2O_3 unit" was removed. In both cases, many new types of oxygen environments are created that exhibit NMR parameters in poor agreement with the signals in the ^{17}O STMAS spectrum (see Figure S5.1). This suggests that if such vacancies are present in bulk γ-Al_2O_3, these are most likely at very low levels.

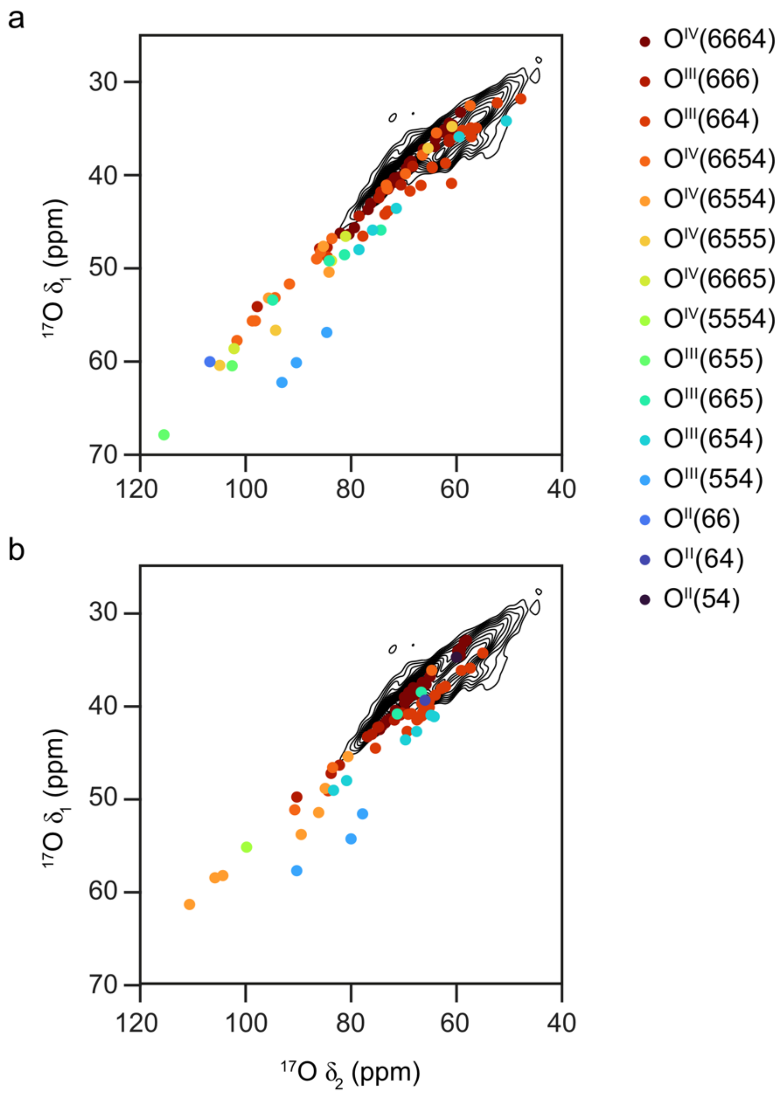

Figure S5.1. Plots showing the overlay of (δ_1, δ_2) centre-of-gravity shifts predicted (using DFT) for different O environments in two models (Models SPV1-2 in (a) and (b), respectively) of bulk γ-Al_2O_3 generated from Model SP6 by removing three O^{2-} and two Al^{3+} cations, with the ^{17}O (20.0 T, 30 kHz) DQF-STMAS spectrum of γ-$Al_2{}^{17}O_3$(950 °C) from Figure 2b of the main text.

S6. Structural models of surfaces of γ-Al_2O_3

Figure S6.1 shows the three main surfaces of the cubic spinel unit cell of γ-Al_2O_3 (shown without any cation vacancies), corresponding to the (100), (110) and (111) planes. Unlike the (100) and (100) planes, normal to the (111) planes there are alternating layers of O^{2-} and Al^{3+} ions. When cation vacancies are considered, this leads to a significantly larger number of inequivalent cleavages that could be performed.

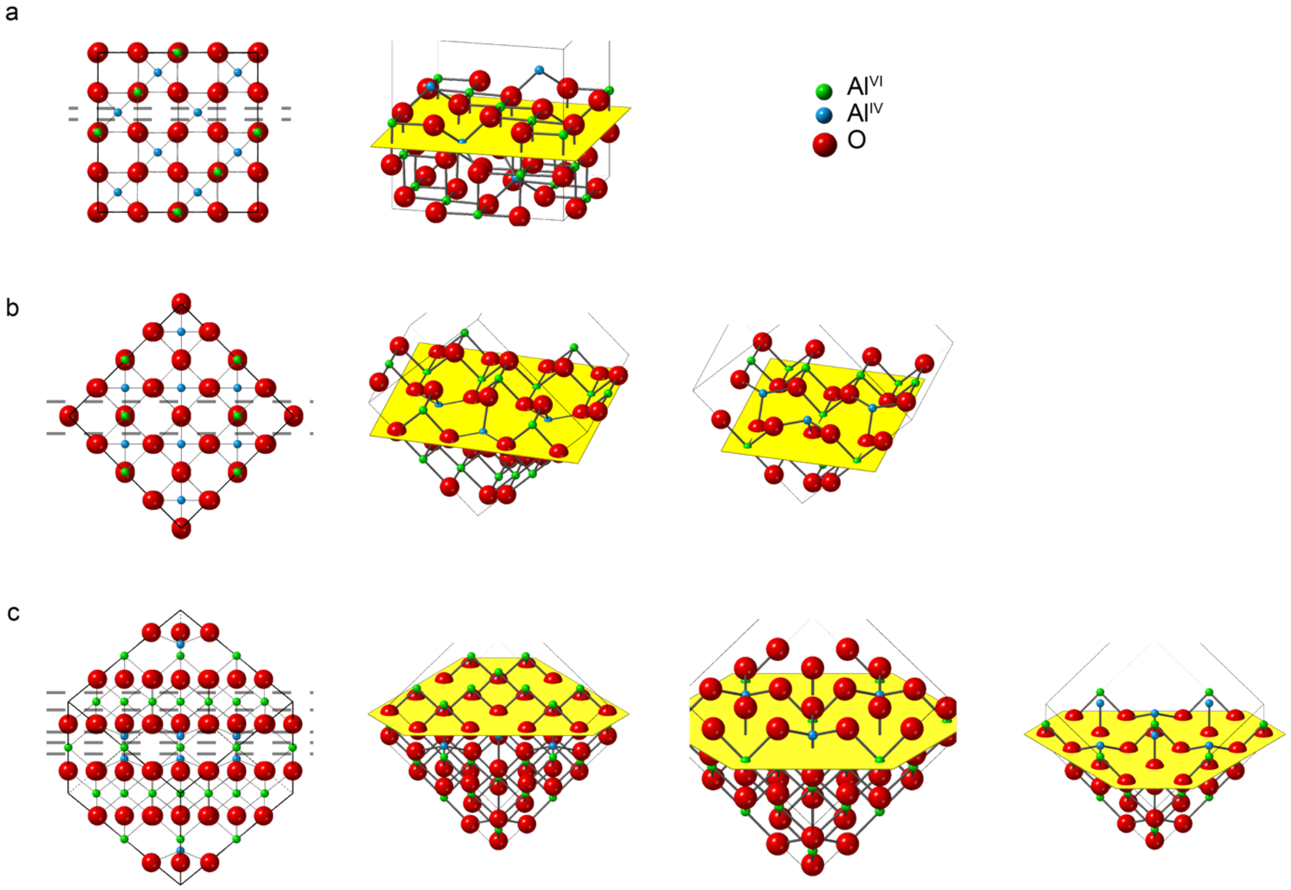

Figure S6.1. Schematic showing the three different surfaces of γ-Al_2O_3 (generated from the cubic spinel cell and shown here without any cation vacancies), corresponding to the (a) (100), (b) (110) and (c) (111) planes.

Structural models for DFT calculations of the γ-Al_2O_3 surface were generated by cleaving the bulk models of γ-Al_2O_3 along the (100), (110) and (111) planes at the top and bottom of a slab. These were subsequently hydroxylated on both sides by adding protons and hydroxyl groups. Slabs were separated by a vacuum gap of 15 Å to avoid any unphysical interaction

between periodic images. Models SP6-8 were cleaved along the unit cell *a* axis (giving rise to (100), (110) and (111) surfaces respectively. Slabs of thickness 17-18 Å were generated (corresponding to 10 oxide layers for structures with (100) and (110) surface planes and 7-8 for structures with (111) surface planes. For two structures, an additional layer of 5-6 water molecules were inserted on both sides of the slab. From the 22 fully hydroxylated structures, 14 partially hydroxylated models were generated by removing as many protons and hydroxyl groups as possible without directly generating Al sites with coordination numbers lower than four and maintaining appropriate charge balance prior to geometry optimization. In most cases, this optimisation was performed in two steps. First, the fractional coordinates of the ions in the inner layers of the slab were constrained, and in the second step, all ionic positions were allowed to relax. The unit cell parameters were not allowed to change during geometry optimisation and were fixed to the values obtained for the bulk structures, with the exception of the unit cell axis *a*, along which the vacuum region was inserted. Figure S6.2 shows an example slab structure used for the computational modelling.

The model codes denote these are slab (SL) structures, cleaved along the (hkl) planes, and whether fully (F) or partially (P) hydroxylated and whether water has been added (W)

e.g.,

SL111_1_F	Slab model 1 cleaved along the (111) planes, fully hydroxylated
SL100_3_P	Slab model 3 cleaved along the (100) planes, partially hydroxylated
SL110_8_F_W	Slab model 8 cleaved along the (110) planes, fully hydroxylated with water added

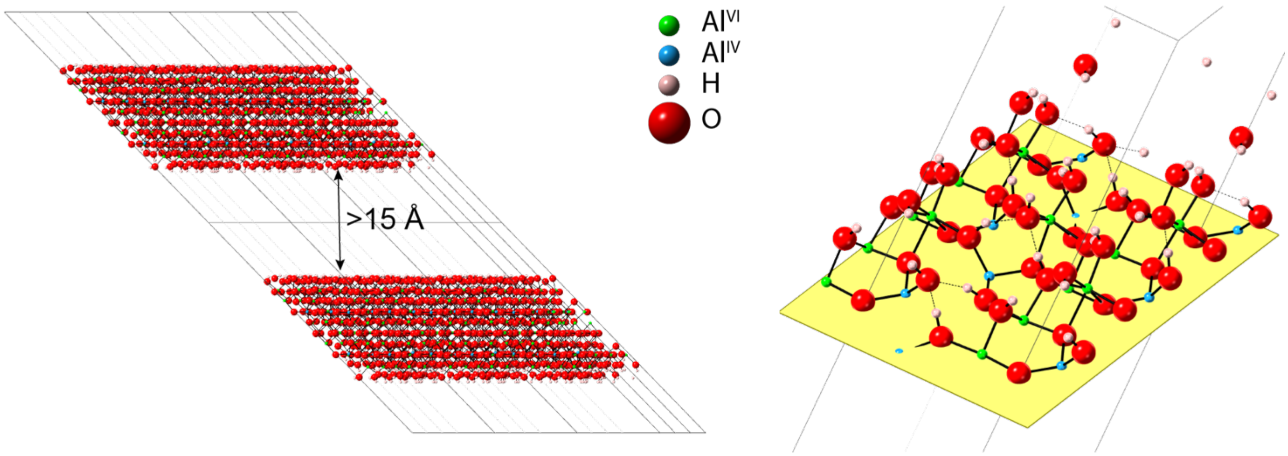

Figure S6.2. (a) Schematic showing a slab structure used for computational modelling of the surface structure of γ-Al_2O_3, with vacuum inserted to ensure sufficient separation of the slabs. (b) Expansion showing the hydroxylated surface.

Figure S6.3 shows the overlay of DFT-predicted ^{17}O NMR parameters for O environments in different layers (1-5 from the surface) of the slab models of the γ-Al_2O_3 described above. Only (δ_1, δ_2) centre-of-gravity shifts for non-protonated O are shown overlaid on the ^{17}O STMAS spectrum of γ-$Al_2{}^{17}O_3$(950 °C), but δ_2 centre-of-gravity shifts for all O are overlaid on the ^{17}O MAS spectrum of γ-$Al_2{}^{17}O_3$(500 °C).

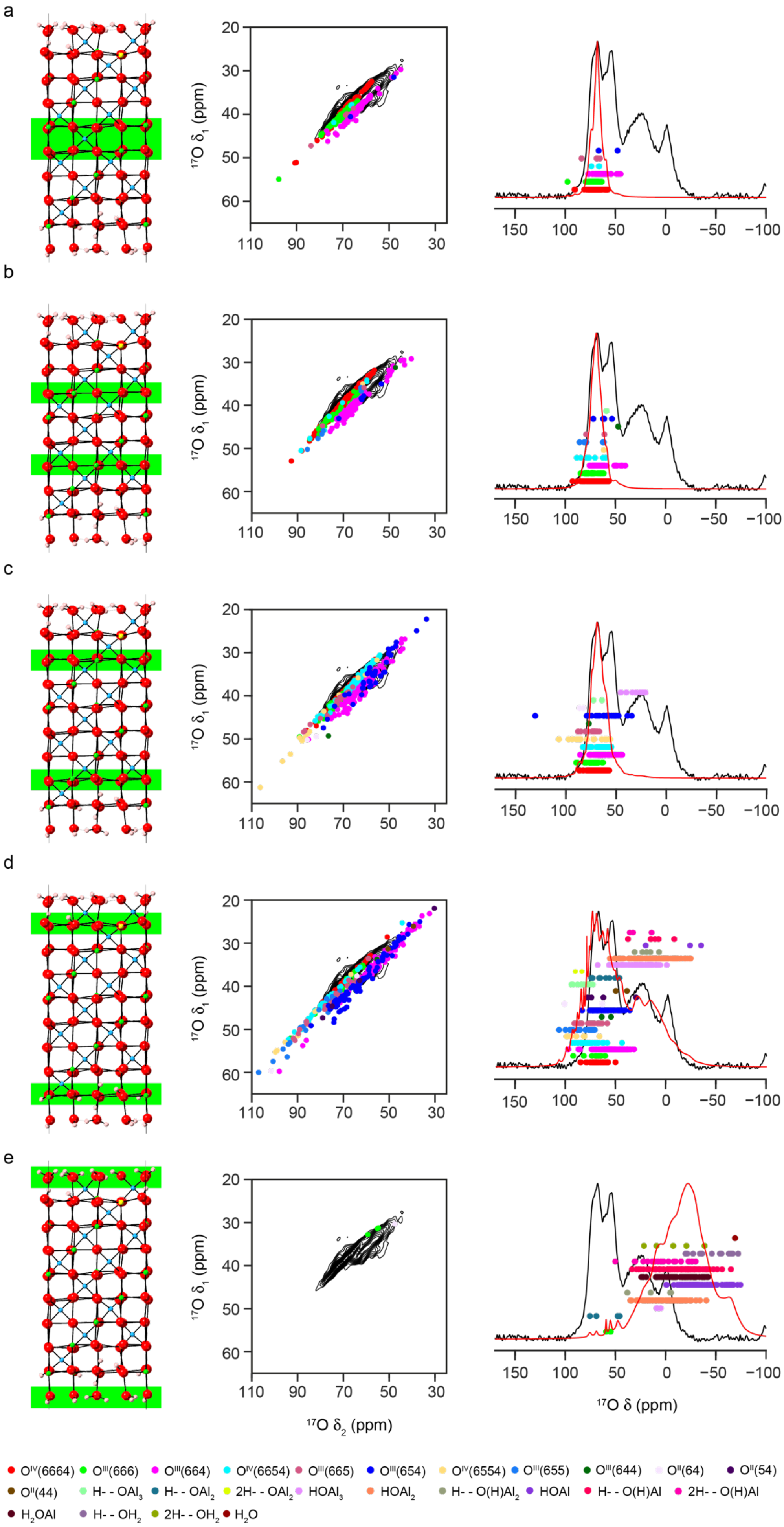
a
b
c
d
e
^{17}O δ_1 (ppm)
^{17}O δ_2 (ppm)
^{17}O δ (ppm)
O^{IV}(6664)
O^{III}(666)
O^{III}(664)
O^{IV}(6654)
O^{III}(665)
O^{III}(654)
O^{IV}(6554)
O^{III}(655)
O^{III}(644)
O^{II}(64)
O^{II}(54)
O^{II}(44)
H- - OAl_3
H- - OAl_2
2H- - OAl_2
$HOAl_3$
$HOAl_2$
H- - $O(H)Al_2$
HOAl
H- - O(H)Al
2H- - O(H)Al
H_2OAl
H- - OH_2
2H- - OH_2
H_2O

Figure S6.3. Plots showing predicted NMR parameters for the slab models of γ-Al_2O_3, as a function of the number of layers from the surface. The (δ_1, δ_2) centre-of-gravity shifts for only non-protonated O are shown overlaid on the ^{17}O (20.0 T, 30 kHz) DQF-STMAS spectrum of γ-$Al_2{}^{17}O_3$(950 °C) from Figure 2b of the main text. The δ_2 centre-of-gravity shifts for all O are shown overlaid on the ^{17}O (20.0 T, 20 kHz) MAS NMR spectrum of γ-$Al_2{}^{17}O_3$(500 °C) from Figure 2a of the main text.

S7. ^{17}O MAS and CP MAS NMR of γ-Al_2O_3

Figure S7.1 shows ^{17}O MAS and CP MAS NMR spectra of γ-$Al_2{}^{17}O_3$(500 °C) at 20.0 T and 14.1 T. The spectra contain two signals that result primarily from protonated O species, which are resolved at 20.0 T (a broad resonance at 20 ppm and a sharp signal at –2 ppm), but are overlapped at 14.1 T. Only the broader signal is seen in the CP experiments, reflecting the faster relaxation as a result of surface dynamics for the sharp signal. As the contact time is further increased CP transfer to the non-protonated O species is seen, although it can be noted that the intensity of the signal at higher δ_2 is relatively lower, reflecting the lack of O^{IV}(6664) environments in the surface and sub surface layers.

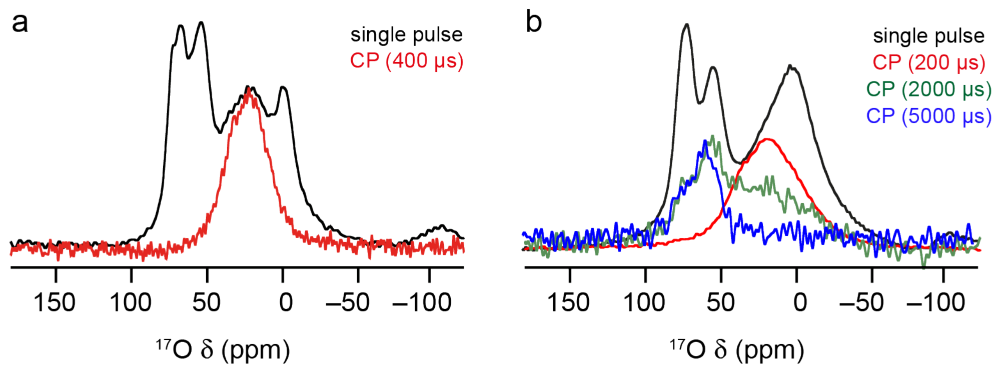

Figure S7.1. ^{17}O MAS and CP MAS NMR spectra of γ-$Al_2{}^{17}O_3$(500 °C) at (a) 20.0 T and (b) 14.1 T, with MAS rates of 20 kHz and 14 kHz, respectively.

Spectra are the result of averaging 4152 (MAS) and 10240 (CP MAS) transients with a recycle interval of 0.5 s. Contact times, τ_{CP}, are given in the figure. CP MAS spectra are shown normalised but are arbitrarily scaled relative to the MAS spectrum.

Figure S7.2 compares ^{17}O MAS (20.0 T, 20 kHz) NMR spectra of γ-$Al_2{}^{17}O_3$(500 °C) just after ^{17}O enrichment and after being stored for 9 months. The intensity of the sharp signal from protonated O species decreases with storage time (likely due to back exchange with $H_2{}^{16}O$ from the air), with the decrease being less for the portion of the sample kept in a rotor and more for the portion stored within a sample vial and accessed on a number of occasions.

Note that there is also a decrease in intensity of the broader signal from protonated O species over this time period, but this is less pronounced.

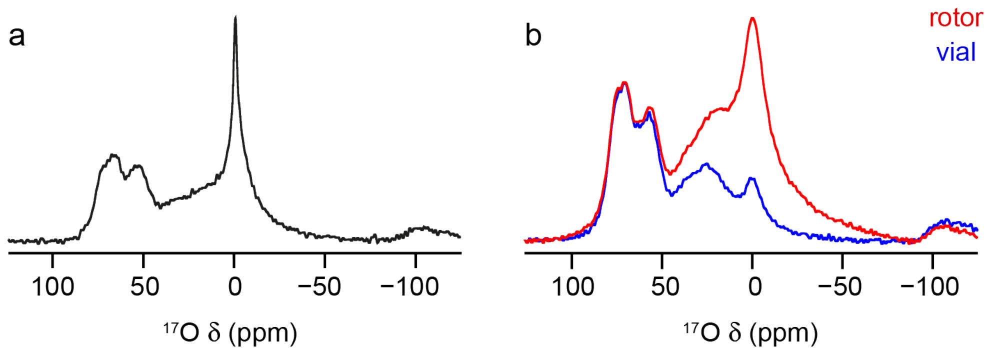

Figure S7.2. ^{17}O (20.0 T, 20 kHz) MAS NMR spectra of γ-$Al_2{}^{17}O_3$(500 °C) (a) 2 days after ^{17}O enrichment and immediate packing in a rotor, (b) after 9 months storage in either the rotor (red) or in a vial that had been opened on multiple occasions (blue). Spectra are the result of averaging 2048 transients with a 0.5 s recycle interval. In (b), spectra are shown normalised to the intensity of the most deshielded peak.

Although the two resonances from protonated O in the ^{17}O MAS spectra of γ-$Al_2{}^{17}O_3$(500 are not resolved at 14.1 T, the broad resonance resulting from the less mobile aluminols in the sub surface layer may be selectively observed in echo-based experiments owing to the longer T_2 relaxation time.

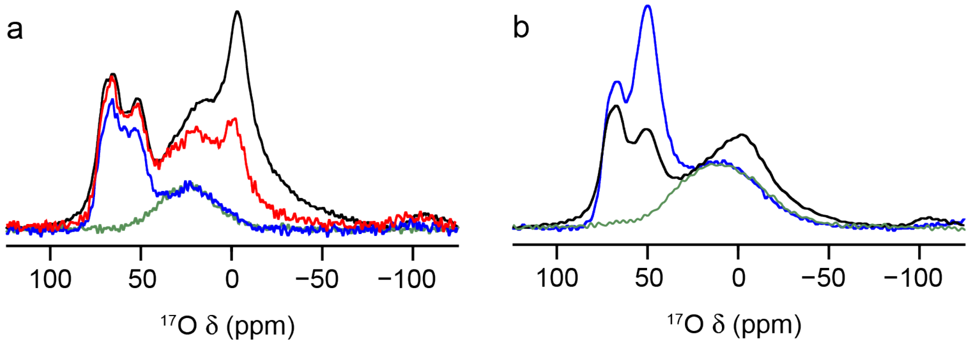

Figure S7.3. ^{17}O MAS NMR spectra of γ-$Al_2{}^{17}O_3$(500 °C) at (a) 20.0 T and 20 kHz MAS and (b) 14.1 T and 14 kHz MAS. In (a), spectra have been acquired using a single pulse (black), spin echo (red and blue, with echo intervals of 50 and 250 μs, respectively) and CP (green, with τ_{CP} of 100 μs). Spectra are the result of averaging 2048 (MAS), 4096 (spin echo) and 2752 (CP) transients with a 0.5 s recycle interval. In (b), spectra have been acquired using a single pulse (black), spin echo (blue, with an echo interval of 71.4 μs), and CP (green, with τ_{CP} of 200 μs). Spectra are the result of averaging 3072 (MAS), 5120 (spin echo) and 5120 (CP) transients with a 0.5 s recycle interval. In (a), the two spin echo NMR spectra are shown with the correct relative intensities, but MAS and CP MAS spectra are shown with arbitrary intensity to facilitate the comparison of the spectral lineshapes. In (b), all spectra are shown with arbitrary intensity to facilitate the comparison of the spectral lineshapes.

The computational results suggest that the broader resonance attributed to protonated O species in the ^{17}O MAS NMR spectrum of γ-$Al_2{}^{17}O_3$(500 °C) arises from aluminols. However, it is possible that this signal could, alternatively, be assigned to less mobile or strongly hydrogen bonded water molecules. These two types of signals could be distinguished using ^{17}O-^{27}Al dipolar dephasing measurements, with the latter species much further from Al^{3+} cations. Figure S7.4 shows ^{17}O MAS NMR spectra acquired with ^{27}Al TRAPDOR dipolar recoupling of varying duration, using the pulse sequence shown. A ^{17}O spin echo was preceded by a hyperbolic secant (HS) pulse for signal enhancement. In the reference spectra (S_0) no pulses are applied to ^{27}Al, while in the S spectra rotor synchronised ^{27}Al (~80 kHz nutation frequency) dipolar recoupling is applied during the echo periods. The dephased difference spectra contain two signals from non-protonated O species and the broader

signal from the protonated O, confirming the assignment of the latter to aluminols rather than to hydrogen-bonded water.

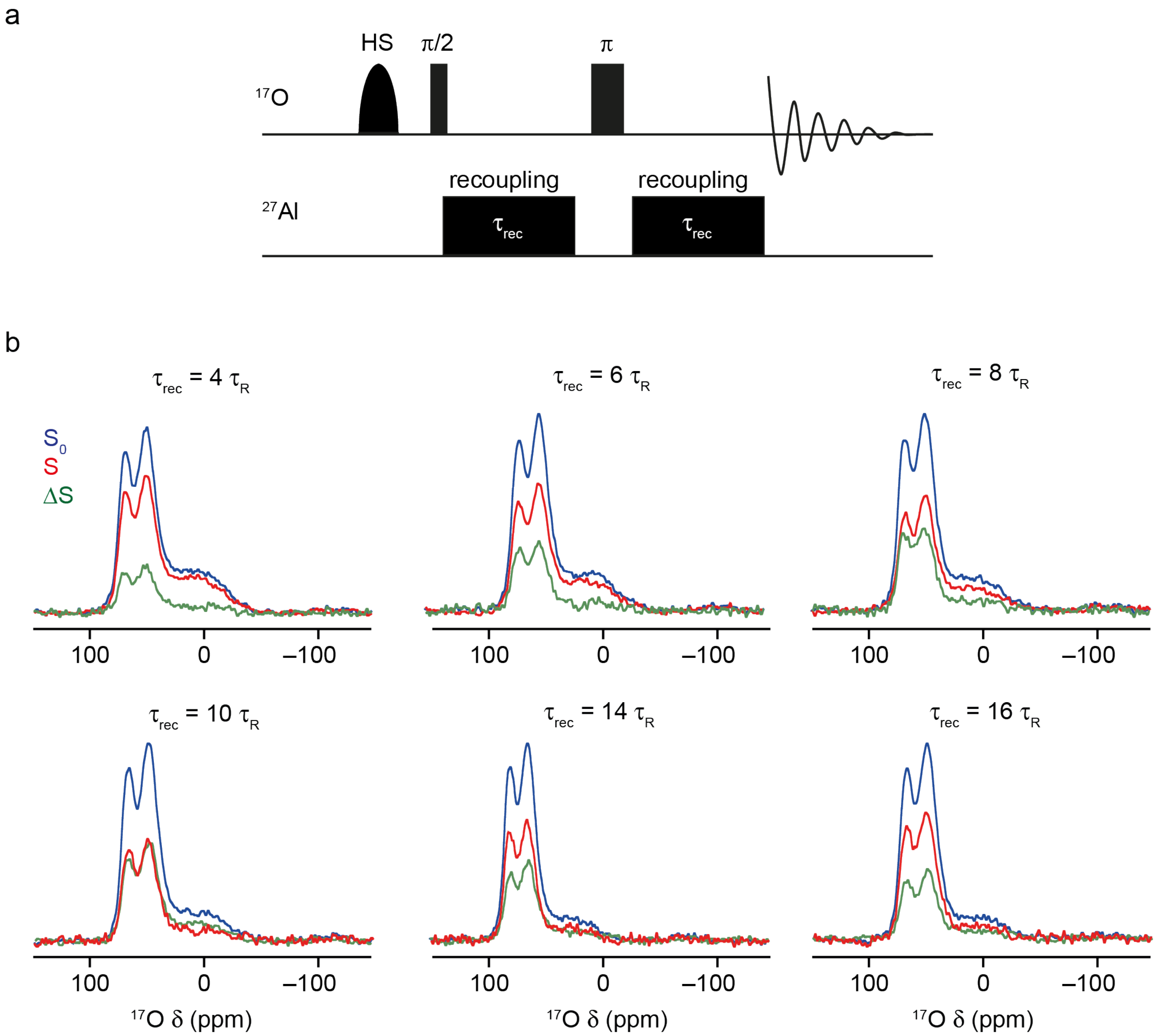

Figure S7.4. (a) Schematic pulse sequence used for ^{17}O-^{27}Al TRAPDOR experiments. (b) ^{17}O (14.1 T, 14 kHz) MAS NMR spectra of γ-$Al_2{}^{17}O_3$(500 °C) with (red) and without (blue) ^{27}Al TRAPDOR pulses (at the recoupling times indicated). The TRAPDOR difference spectra ($\Delta S = S_0 - S$) are shown in green. Spectra are the result of averaging 4224 transients with a recycle interval of 1 s. The HS pulse duration was 1.1 ms.

S8. ^{27}Al MAS and MQMAS NMR of γ-Al_2O_3

Figure S8.1 shows ^{27}Al MAS and MQMAS NMR spectra of γ-Al_2O_3 acquired at 9.4 T and 14.1 T, demonstrating the presence of a distribution of δ_{iso} and P_Q for Al^{IV} and Al^{VI} signals. Table S8.1 gives average values ($<\delta_{iso}>$ and $<P_Q>$) of the NMR parameters extracted from the spectra.

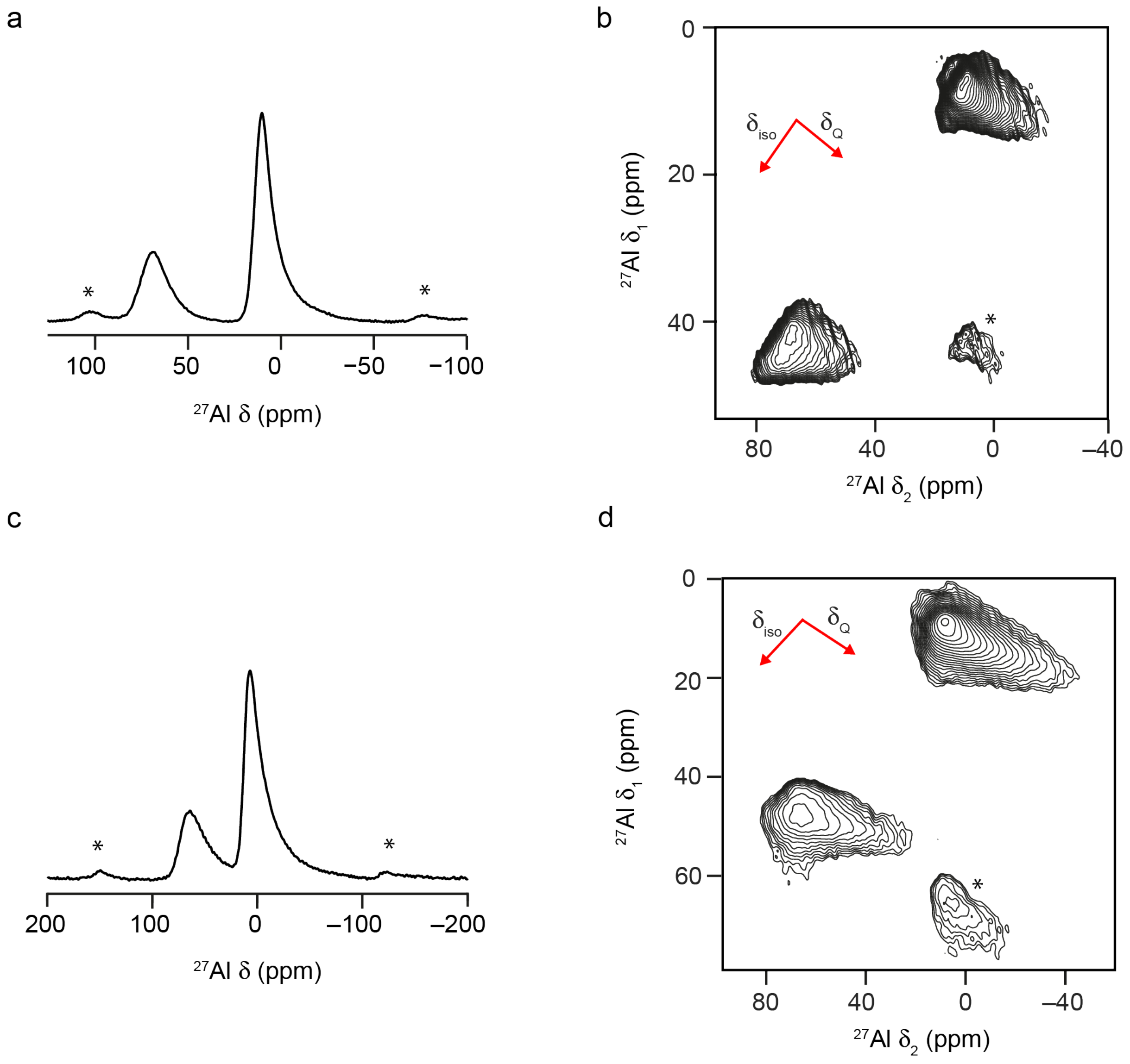

Figure S8.1. ^{27}Al (a, c) MAS and (b, d) MQMAS NMR spectra, acquired at (a, b) 14.1 T and (c, d) 9.4 T, using an MAS rate of 14 kHz. MQMAS spectra were acquired using a z-filtered triple-quantum MAS pulse sequence and are shown after shearing.

Table S8.1. Average ^{27}Al NMR parameters extracted from the spectra in Figure S8.1 and Figure 8c of the main text.

Species	$\langle\delta_{iso}\rangle$ (ppm)	$\langle P_Q\rangle$ / MHz
Al^{IV}	77 (2)	5.0 (4)
A^{V}	37 (3)	4.1 (5)
Al^{VI}	14 (1)	3.5 (5)

S9. ^{27}Al-^{17}O calculated J couplings

Table S9.1 gives the DFT calculated J couplings for the Al species in Model SP7 of bulk γ-Al_2O_3 (shown in Figure S3.5).

Table S9.1. Calculated ^{27}Al-^{17}O J couplings for Model SP7.

Al species	O species	J_{Al-O} / Hz	$\langle J_{Al-O} \rangle$ / Hz	Range J_{Al-O} / Hz
	O^{IV}	5.1		
	O^{IV}	5.1		
	O^{IV}	5.5		
Al^{VI}	O^{IV}	8.7	6.35	7.1
	O^{IV}	10.4		
	O^{III} (3)	3.3		
	O^{IV}	2.0		
	O^{IV}	6.8		
	O^{IV}	8.5		
Al^{VI}	O^{III} (2)	1.4	4.33	7.2
	O^{III} (2)	1.3		
	O^{III} (3)	6.0		
	O^{IV}	7.3		
	O^{IV}	7.3		
Al^{IV}	O^{III} (2)	19.5	13.4	12.2
	O^{III} (2)	19.5		
	O^{IV}	10.2		
	O^{IV}	11.4		
Al^{IV}	O^{IV}	12.6	13.65	10.2
	O^{III} (2)	20.4		

S10. ^{1}H MAS NMR of γ-Al_2O_3

Figure S10.1 shows the change to the ^{1}H MAS NMR spectrum (acquired with an MAS rate of 40 kHz) of dehydrated γ-Al_2O_3 when its slowly rehydrates inside the NMR rotor. This leads to increased intensity at higher shift, in agreement with the differences in spectral lineshapes seen in the main text (Figure 9a) for γ-Al_2O_3 dehydrated at different temperatures.

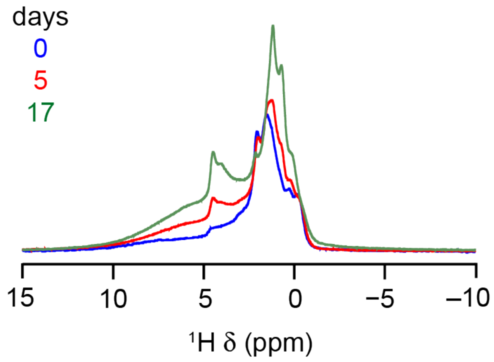

Figure S10.1. ^{1}H (14.1 T, 40 kHz) MAS NMR spectra of dehydrated γ-Al_2O_3 upon gradual rehydration inside a 1.9 mm rotor. Spectra are acquired at 266 K and are the result of averaging 16, 64 and 64 transients with a recycle intervals of 1 s, 10 s and 3 s, and are shown 0, 5 and 17 days after packing. Spectra are normalised such that the intensity of the most shielded signal is the same in each.

Figure S10.2 shows overlay of DFT-predicted NMR shifts (δ_{iso}) for all H in the fully and partially hydroxylated slab models of γ-Al_2O_3 for μ_1, μ_2 and μ_3 OH groups. In addition to the labelling used in the main text (Figure 9), the coordination number of the aluminium ions bonded to the hydroxyl oxygen is also considered. For example, (HA) μ_2-OH (HD) (Al-46) denotes a bridging hydroxyl where the proton is a hydrogen bond donor (HD), the oxygen is hydrogen bond acceptor (HA) and the OH groups is bridging between an Al^{IV} and an Al^{VI}.

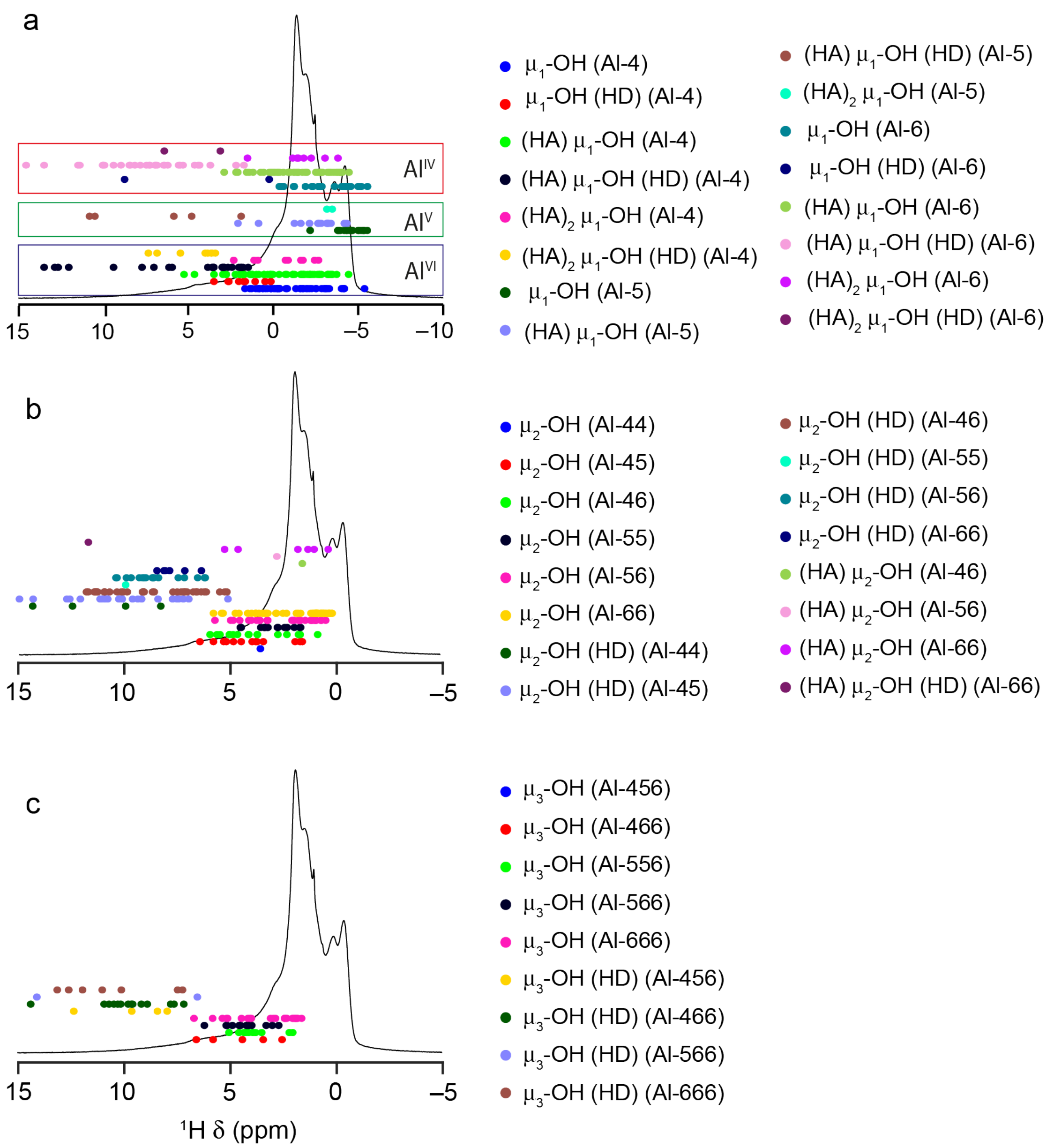

Figure S10.2. Plots showing the overlay of the DFT-predicted NMR shifts (δ_{iso}) for all H in the fully and partially hydroxylated slab models of γ-Al_2O_3, overlayed with the ^{1}H (14.1 T, 20 kHz) DEPTH MAS NMR spectrum of γ-Al_2O_3 dehydrated at 550 °C from Figure 9b of the main text.

^{1}H-^{27}Al RESPDOR experiments were carried out using the pulse sequence shown in Figure S10.3a, with R12$_3^5$ recoupling incremented in steps of τ_R, at 40 kHz MAS. The saturation pulse applied to ^{27}Al had a duration of 1.5 τ_R at 100 kHz rf amplitude. The RESPDOR fraction is defined as $(S_0 - S)/ S_0$, where the saturation pulse is present for S and missing for S_0. For analysis, the ^{1}H MAS NMR spectrum was decomposed into 10 separate Voigt components, as shown in Figure S10.3b. These signals should not be regarded as spectral signals from

individual distinct ^{1}H, but rather as a component that describes the distribution of δ_{iso} corresponding to ^{1}H in similar environments. Upon fitting each experimental spectrum, only the intensity of components 1-9 was varied, but it was necessary to allow the width and position of component 10 to vary (as a result of the low spectral intensity in this region). This had very little effect on the intensities of the remaining signals 1-9. Component 10 was, therefore, excluded from the final analysis.

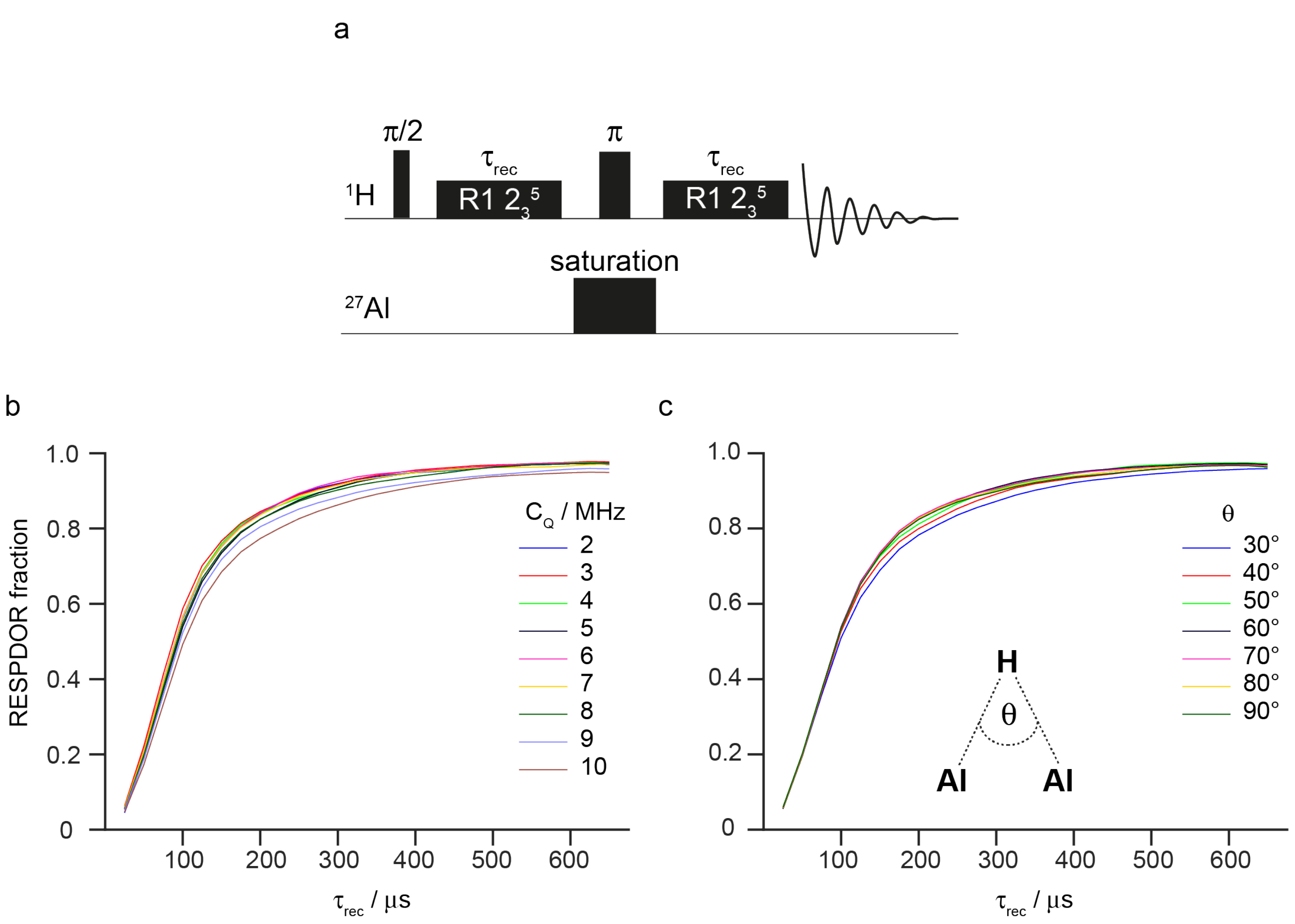

Figure S10.3. (a) Schematic pulse sequence used for the ^{1}H-^{27}Al RESPDOR experiments. (b, c) Plots showing the dependence of the RESPDOR fraction (determined by numerical simulations for the H_2Al spin system shown in Figure 10c of the main text) on (b) the ^{27}Al C_Q and (c) the angle, θ, between the two H-Al internuclear vectors. In (b) θ = 60°, while in (c) ^{27}Al C_Q = 5 MHz, and in both cases $d_1 = d_2 = 2.4$ Å.

RESPDOR fractions were simulated using SIMPSON, for a $^{1}H(^{27}Al)_2$ spin system, with H-Al distances d_1 and d_2 determining the two different heteronuclear dipolar couplings. No ^{1}H-^{1}H couplings were included as these were expected to be small under the recoupling scheme

and high MAS rate used. For the simulations shown in the main text the ^{27}Al C_Q was fixed at 5 MHz and (when relevant) the angle between the two H-Al internuclear vectors fixed at 60°. Variation of these parameters has only a small effect on the calculated RESPDOR fraction, as shown in Figure S10.3. In each simulation, 11 γ_{MR} angles and 100 (α_{MR}, β_{MR}) angle pairs distributed according to the REPULSION scheme were used.

Figure S10.4 shows ^{1}H DQ MAS spectra (acquired using BABA recoupling times of 25 μs and 100 μs), along with cross sections taken parallel to δ_2 at the positions shown).

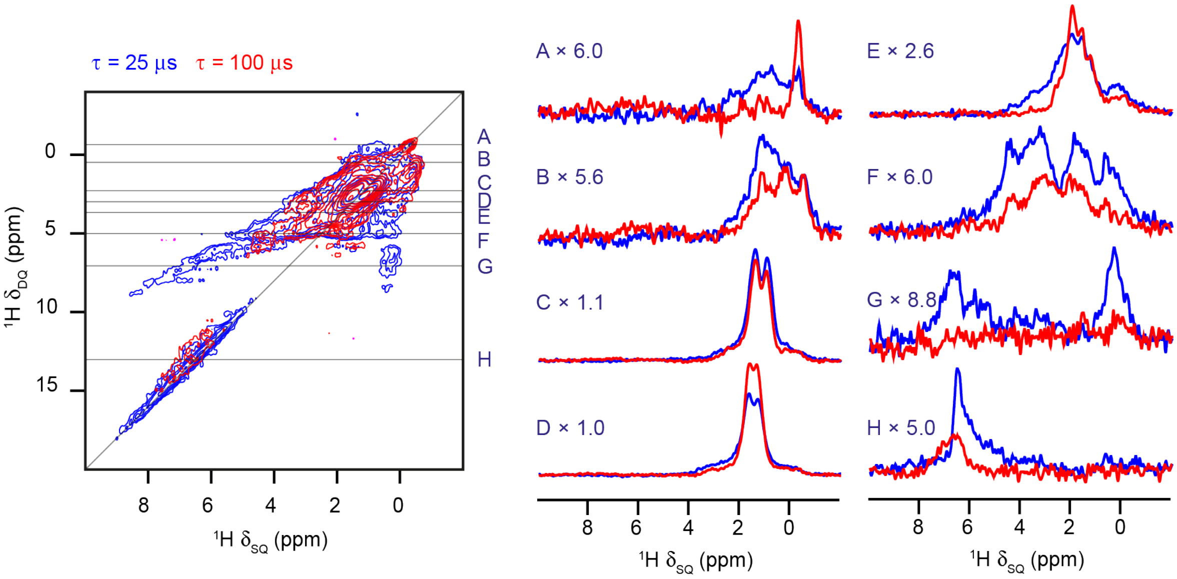

Figure S10.4. ^{1}H (14.1 T, 40 kHz) DQ MAS spectra, acquired using BABA recoupling times of 25 μs (blue) and 100 μs (red), along with cross sections taken parallel to δ_2 at the positions shown. Spectra are the result of averaging 80 transients for each of 200 t_1 increments of 25 μs, with a recycle interval of 1 s.

S11. References

S1. Kovarik, L.; Bowden, M.; Genc, A.; Szanyi, J.; Peden, C. H. F.; Kwak, J. H. Structure of δ-Alumina: Toward the Atomic Level Understanding of Transition Alumina Phases. *J. Am. Ceram. Soc.* **2014**, *118*, 18051-18058.

S2. Kovarik, L.; Bowden, M.; Shi, D.; Washton, N. M.; Andersen, A.; Hu, J. Z.; Lee, J.; Szanyi, J.; Kwak, J. H.; Peden, C. H. F. Unravelling the Origin of Structural Disorder in High Temperature Transition Al_2O_3: Structure of θ-Al_2O_3. *Chem. Mater.* **2015**, *27*, 7042-7049.

S3. Paglia, G. Determination of the Structure of γ-Alumina using Empirical and First-Principles Calculations Combined with s Experiments. PhD Thesis, Curtin University of Technology, 2004.
